# Recent Development of Gas Sensing Platforms Based on 2D Atomic Crystals

**DOI:** 10.34133/2021/9863038

**Published:** 2021-04-21

**Authors:** Jiacheng Cao, Qian Chen, Xiaoshan Wang, Qiang Zhang, Hai-Dong Yu, Xiao Huang, Wei Huang

**Affiliations:** ^1^Frontiers Science Center for Flexible Electronics, Xi'an Institute of Flexible Electronics (IFE), and Xi'an Institute of Biomedical Materials & Engineering, Northwestern Polytechnical University, 127 West Youyi Road, Xi'an 710072, China; ^2^Key Laboratory of Flexible Electronics (KLOFE) & Institute of Advanced Materials (IAM), Nanjing Tech University (NanjingTech), 30 South Puzhu Road, Nanjing 211800, China

## Abstract

Sensors, capable of detecting trace amounts of gas molecules or volatile organic compounds (VOCs), are in great demand for environmental monitoring, food safety, health diagnostics, and national defense. In the era of the Internet of Things (IoT) and big data, the requirements on gas sensors, in addition to sensitivity and selectivity, have been increasingly placed on sensor simplicity, room temperature operation, ease for integration, and flexibility. The key to meet these requirements is the development of high-performance gas sensing materials. Two-dimensional (2D) atomic crystals, emerged after graphene, have demonstrated a number of attractive properties that are beneficial to gas sensing, such as the versatile and tunable electronic/optoelectronic properties of metal chalcogenides (MCs), the rich surface chemistry and good conductivity of MXenes, and the anisotropic structural and electronic properties of black phosphorus (BP). While most gas sensors based on 2D atomic crystals have been incorporated in the setup of a chemiresistor, field-effect transistor (FET), quartz crystal microbalance (QCM), or optical fiber, their working principles that involve gas adsorption, charge transfer, surface reaction, mass loading, and/or change of the refractive index vary from material to material. Understanding the gas-solid interaction and the subsequent signal transduction pathways is essential not only for improving the performance of existing sensing materials but also for searching new and advanced ones. In this review, we aim to provide an overview of the recent development of gas sensors based on various 2D atomic crystals from both the experimental and theoretical investigations. We will particularly focus on the sensing mechanisms and working principles of the related sensors, as well as approaches to enhance their sensing performances. Finally, we summarize the whole article and provide future perspectives for the development of gas sensors with 2D materials.

## 1. Introduction

Acting as an indispensable component in the era of the Internet of Things, gas sensors have been intensively studied and applied in a broad range of fields including gas emission control, agricultural and industrial production, military defense, environmental safety, and medical diagnostics [1–3]. High sensitivity, good selectivity, and rapid response/recovery time, as well as ambient working conditions, are the main requirements for high-performance gas sensors.

The gold standard for gas analysis has been spectroscopy-based techniques, typically including gas chromatography (GC), infrared (IR) absorption spectroscopy, and Raman scattering spectroscopy. GC is able to perform multicomponent analysis by separating different gas components in the chromatographic column according to their distribution coefficients between the mobile phase (i.e., the carrier gas) and the stationary phase (e.g., a solid adsorbent or liquid support) [4]. Separated gas components will successively enter a detector that converts the component signals to electric signals by various means, such as the thermal conductivity detector [5] and flame ionization detector [6]. Owing to the limited detection capability of these detectors, mass spectrometry and optical spectroscopies have been coupled with GC to obtain better qualitative and quantitative information [7–10]. The IR absorption spectroscopy is based on the selective adsorption of laser radiation of gas molecules, mostly in the middle-infrared wavelength, which can induce the change of the dipole moment of gas molecules, reflected in the position and intensity of their adsorption spectra; different gas molecules usually have their own characteristic spectral lines like “fingerprints” showing both the qualitative and quantitative information. However, the IR spectroscopy still has some limitations, such as a restricted selectivity, especially being inactive to diatomic homonuclear molecules such as nitrogen, oxygen, and hydrogen [11]. The Raman scattering spectroscopy, which involves the inelastic scattering of photons upon interaction with gas molecules, is capable of providing fingerprints for a wide range of gas molecules including those inactive to IR adsorption spectroscopy [12]. However, due to the relatively weak signal of the Raman scattering spectroscopy, higher power laser sources or cavity-enhanced Raman spectroscopy techniques are needed for precise detection [13].

The abovementioned spectroscopic sensing techniques, despite their robustness, usually require expensive and bulky instruments and thus are not easily accessible and not suitable for applications that require on-site investigation and in situ monitoring. Alternative gas sensing techniques are therefore being developed in parallel for reduced cost and better portability. These gas sensors include electrical sensors, such as electrochemical sensors [14], chemiresistors [15], field-effect transistors (FETs) [16], Schottky diodes [17], conductometric (or chemiresistive) sensors [18], and impedance sensors [19], and optical sensors, such as fiber optic gas sensors [20] and photonic crystal gas sensors [21]. Although conventional gas sensing materials such as metal oxides, conducting polymers, and carbon nanotubes have long been developed and applied in commercial gas sensors, they still suffer from problems such as limited selectivity, poor reproducibility, and, in most cases, high operating temperatures [22–24]. Therefore, the exploration and development of alternative gas sensing materials require continuous efforts.

**Figure 1:**
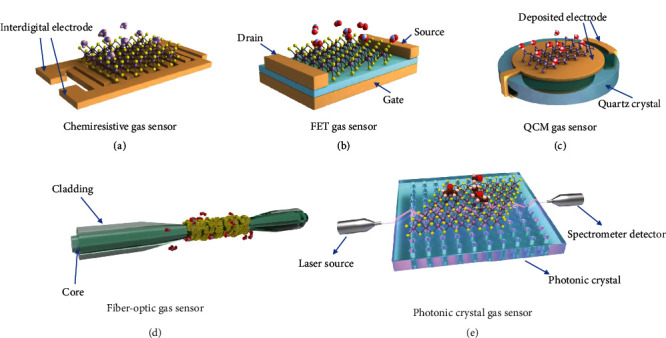
Schematic illustration of (a) a chemiresistive gas sensor, (b) a bottom-gated FET sensor, (c) a quartz crystal microbalance (QCM) gas sensor, (d) a fiber optic gas sensor, and (e) a photonic crystal gas sensor involving 2D materials.

Two-dimensional (2D) atomic crystals, such as metal chalcogenides (MCs), black phosphorus (BP), and MXenes, are considered attractive candidates for the fabrication of gas sensors due to their ultrahigh specific surface areas with massive reactive sites for interaction with gas molecules, thickness-dependent physical and chemical properties, tunable surface functionalities, solution phase production for scalable device fabrication, and ability to assemble into three-dimensional (3D) architectures with controllable porosities [25–29]. 2D materials have been mostly explored in the form of a chemiresistive gas sensor (Figure 1(a)), field-effect transistor (FET) (Figure 1(b)), quartz crystal microbalance (QCM) (Figure 1(c)), fiber optic gas sensor (Figure 1(d)), and photonic crystal gas sensor (Figure 1(e)). Their basic working principles are described as follows.

Chemiresistive gas sensors have a relatively simple configuration. Typically, sensing materials, such as 2D materials, are cast as a film to connect two predeposited electrodes or on top of an interdigitated electrode (Figure 1(a)). The adsorption of target gas molecules can lead to resistance change of the sensing film, mostly through the exchange of charge carriers. The resistance change, i.e., *S*(%) = [(*R*_g_ − *R*_0_)/*R*_0_] × 100%, where *R*_0_ is the film resistance in the air or inert gas, and *R*_g_ is the film resistance when exposed to the target gas at equilibrium, reflects the sensitivity of the sensor.

Resembling the chemiresistive gas sensors, FET gas sensors also respond to gas adsorption via resistance change (or conductance change) of the sensing channel, which connects the source and drain electrodes (Figure 1(b)). Its advantage is that the sensing response can be tuned by adjusting the gate bias which controls the carrier concentration in the channel and thus tunes the amount of charge carriers in exchange with the absorbed gas molecules. More details will be given in Section 2.1.1.

A quartz crystal microbalance (QCM), typically consisting of a quartz crystal sandwiched between two parallel metal electrodes, is capable of providing extremely sensitive mass measurement in the scale of nano- to microgram per unit area (cm^2^). A QCM makes use of the piezoelectric property of the quartz crystal, which oscillates under an applied voltage across the two electrodes. Its resonant frequency decreases upon mass loading. The relationship between the frequency shift (Δ*f*) and the mass load (Δ*m*) is described by the Sauerbrey equation, Δ*f* = −2.26 × 10^−6^*f*_0_^2^(Δ*m*/*A*), where *f*_0_ is the intrinsic resonant frequency of the piezoelectric crystal and *A* is the surface area of the circular electrode [30]. For gas sensing, the mass loading (or stress) induced by the gas adsorption on the electrode surface is transferred to the quartz crystal below to induce its frequency change (Figure 1(c)). Depositing 2D materials onto the surface of the electrode can help increase the amount of gas adsorption sites and improve the selectivity of the QCM gas sensor [31–33].

One of the most common optical gas sensors is the fiber optic type as shown in Figure 1(d). Its main component is a waveguide consisting of a fiber core and the surrounding cladding. The core has a higher refractive index than the cladding, allowing the light to travel along with the fiber core via total reflection; a small portion of the light is transmitted as evanescent waves perpendicular to the fiber axis with intensity reduced exponentially in the cladding [20]. The adsorption of gas molecules on the cladding changes its refractive index, resulting in the change of the output light signals such as its intensity and wavelength. However, gas adsorption-induced change in the material refractive index is limited in selectivity, and therefore, the cladding is usually functionalized with molecules or nanomaterials, such as 2D materials, which have a specific affinity toward the target gas.

Another similar type of optical gas sensor is the photonic crystal (PC) gas sensor Figure 1(e), where the abovementioned optic fiber is replaced with PCs, which are, mostly, artificial optical materials with periodic changes in the refractive index [34]. When the fiber cladding has a higher refractive index than the PC core with air channels, according to the photonic bandgap theory, light with frequency in the range of the PC bandgap will be bound in and transmit along the air channel with low energy loss [21]. The air channels also serve as cells for the introduction of target gas molecules, which change the refractive index of the PC core and thus change the output light. Functionalization of the PCs with 2D materials is able to enhance the selectivity of the optical gas sensor, which will be discussed in Section 2.1.4.

The gas adsorption on a 2D material can lead to changes in a number of its properties such as its carrier concentration, carrier mobility, work function, band positions (or redox potentials), and oxidation states, as well as the refractive index. A deep understanding of the processes behind the output changes is of paramount importance in designing and developing novel and advanced sensing materials and devices. This contribution is thus aimed at providing a comprehensive review on the recent development of gas sensors based on 2D materials, discussing their sensing mechanisms, influencing factors, and approaches to enhance the gas sensing performance. As gas sensors based on graphene and related materials have been reviewed previously [35–38], this review will mainly cover gas sensors based on other 2D atomic crystals, such as MCs, MXenes, and BP, as well as their composites/heterostructures.

## 2. Metal Chalcogenides (MCs)

**Figure 2:**
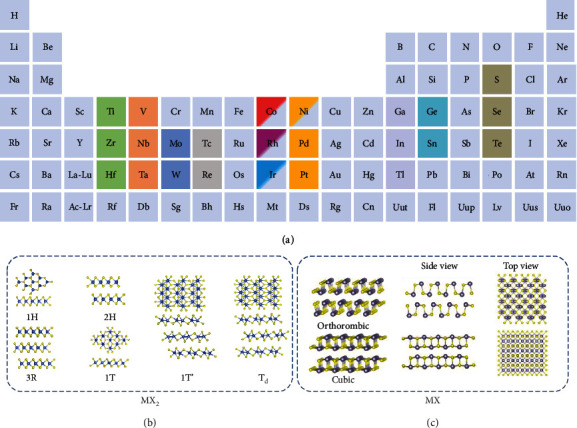
(a) Periodic table highlighting the possible metals and chalcogens to form 2D MCs, drawn based on Ref. [69]. Various crystal structures of transition metal or posttransition metal chalcogenides with the chemical formula of (b) MX_2_ or (c) MX, showing both the top and side views [40], copyright 2018 Science China.

Metal chalcogenides (MCs) in general have a chemical formula of MX_2_ or MX where M is a transition metal such as Nb, Ta, Mo, and W or a posttransition metal such as Sn and In, and X is a chalcogen species such as S, Se, and Te (Figure 2(a)) [39]. They exist in various crystal phases that correlate to their electronic properties (Figure 2(b)) [40]. Most of them are layered structures, and adjacent MX_2_ or MX layers are stacked together via the weak van der Waals forces. Therefore, single- or few-layer MCs can be prepared by top-down methods, such as mechanical cleavage, ultrasonication-assisted liquid-phase exfoliation, and chemical or electrochemical intercalation and subsequent exfoliation [41, 42]. In addition, bottom-up methods, mainly including vapor-based methods such as the physical and chemical vapor deposition (PVD, CVD) and wet chemical methods such as the solvothermal/hydrothermal method, have also been employed to synthesize 2D MCs [43–46]. Due to their attractive chemical, electrochemical, and optoelectronic properties [47–49], MCs are considered promising materials for various applications such as electronic devices [50], catalysis [51], photothermal therapy [52], and energy storage devices [53].

Unlike pristine graphene, which is a gapless semimetal and suffers from poor selectivity when it comes to molecular detection, the tunable band structures of 2D MCs make them attractive candidates for the fabrication of electrical gas sensors [38, 47, 54]. Up till now, not all the MCs have been explored for gas sensing, and the most studied MCs for gas sensing are MoS_2_, WS_2_, MoSe_2_, WSe_2_, ReS_2_, ReSe_2_, SnS_2_, GaS, and GaSe. Whether a MC material is suitable for gas sensing is dependent on a number of factors, such as its bandgap, doping type and level, and surface chemistry. For example, metallic NbS_2_ and VSe_2_ may not be suitable for sensing because of their low electrical resistance and vice versa for the insulating HfS_2_. Other factors that should also be taken into consideration when designing MC-based gas sensing materials, such as the alloying, layer number, and presence of heterojunctions, will be introduced in detail in Sections 2.1 and 2.2. For example, MCs such as MoS_2_ [55] and WS_2_ [56] have been used to detect NO_2_, and MoSe_2_ [57] has been used to sense NH_3_ and ethanol (Table 1). Most MC-based electrical gas sensors are based on the charge transfer mechanism [1, 38, 56, 58–64], while sensing via surface reaction and proton conduction has also been proposed [65–68]. Besides, sensing via gas adsorption-induced change of the refractive index has been explored in MC-functionalized optical sensors [20, 21].

### 2.1. Basic Working Principles

#### 2.1.1. Sensing via Charge Transfer

Gas sensing via charge transfer can be interpreted as electron (or hole) transport between a sensing film and the target gas. Depending on whether the gas is oxidizing (e.g., NO_2_ and SO_2_) or reducing (e.g., NH_3_ and acetone) and the sensing film is an n- or p-type semiconductor, electrons are withdrawn from or donated to the sensing film. For example, mechanical exfoliated n-type MoS_2_ nanosheets showed an increased resistance upon NO_2_ adsorption and decreased resistance under NH_3_ exposure [55], and hydrothermally synthesized p-type WS_2_ nanosheets presented a reduced resistance toward NO_2_ and an opposite response toward NH_3_ [70]. The dopant type of MCs is dependent on their compositions (or alloying), crystal structures, and their preparation methods as shown in Table 1.

**Figure 3:**
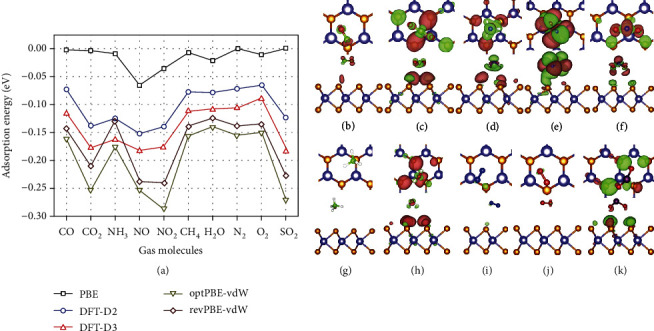
(a) Adsorption energies (eV) of various gas molecules on the MoS_2_ monolayer determined from different methods: PBE, DFT-D2, DFT-D3, optPBE, and revPBE. Isosurface plot of the electron charge density difference for (b) CO, (c) CO_2_, (d) NH_3_, (e) NO, (f) NO_2_, (g) CH_4_, (h) H_2_O, (i) N_2_, (j) O_2_, and (k) SO_2_ on the MoS_2_ monolayer with the isovalue of ±0.0002 e/Bohr^3^ (top view and side view are provided in the first row and second row for each adsorbed molecules). The charge accumulation is represented in pink, and the charge depletion is in lime, respectively [72], copyright 2014 Elsevier Ltd.

The selectivity of this type of sensor largely depends on the ability of the sensing material to bind with the target gas and the tendency to receive or donate electrons toward the gas. For example, experimental studies have shown good selectivity of MoS_2_ sensors toward NO_2_ gas [71], which agrees with theoretical calculation results that the adsorption energies of NO_2_/NO gases on MoS_2_ are generally lower and the amount of electrons transferred is higher as compared to that toward other gases including CO, CO_2_, NH_3_, NO, NO_2_, CH_4_, H_2_O, N_2_, O_2_, and SO_2_ (Figure 3) [72].

In addition to the intrinsic properties of gas adsorption, there are other factors that can influence the sensing response of MC-based gas sensors via charge transfer, which will be discussed in the following context.

**Figure 4:**
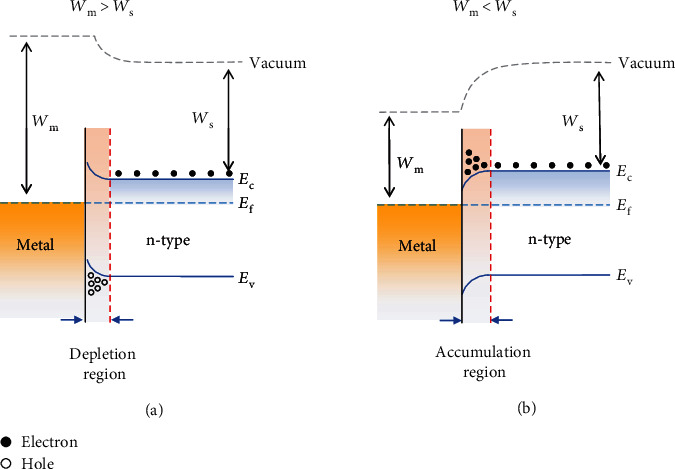
Schematic illustration of the formation of (a) a Schottky contact and (b) an Ohmic contact.

*(1) Electrode Channel Contact*. When a metal and a semiconductor are in contact with each other, either a Schottky barrier or an Ohmic contact is formed depending on the semiconductor type and the relative position of their work functions [73]. Specifically, taking an n-type semiconductor as an example, when its work function *W*_s_ is smaller than that of the electrode (*W*_m_), i.e., *W*_m_ > *W*_s_, the two Fermi levels tend to reach the same level once they are in close contact, and the band of the semiconductor near the interface tends to bend upward, resulting in the formation of a potential barrier called the Schottky barrier (Figure 4(a)). Only electrons with energies higher than the potential barrier can travel across the interface [74], and thus, the current is mainly controlled by the barrier height and width of the depletion layer [75]. When *W*_m_ < *W*_s_, the band of the semiconductor tends to bend downward at the interface where an electron accumulation region could form (Figure 4(b)), leading to an Ohmic contact.

The Schottky barrier present at a semiconductor-metal interface is an important tunable factor influencing its gas sensing performance [76–78]. According to previous studies [79], the *I*-*V* curve of a semiconductor-electrode contact has a basic correlation with the Schottky barrier height:

(1) $$\begin{array}{c} I=I_{0}\;\exp\left(\frac{qV}{nkT}\right)\cdot \left[1-\exp\left(-\frac{qV}{nkT}\right)\right], \end{array}$$

(2) $$\begin{array}{c} I_{0}={SAT}^{2}\exp\;\left(\frac{-{Ø}_{\text{b}}}{kT}\right). \end{array}$$

**Figure 5:**
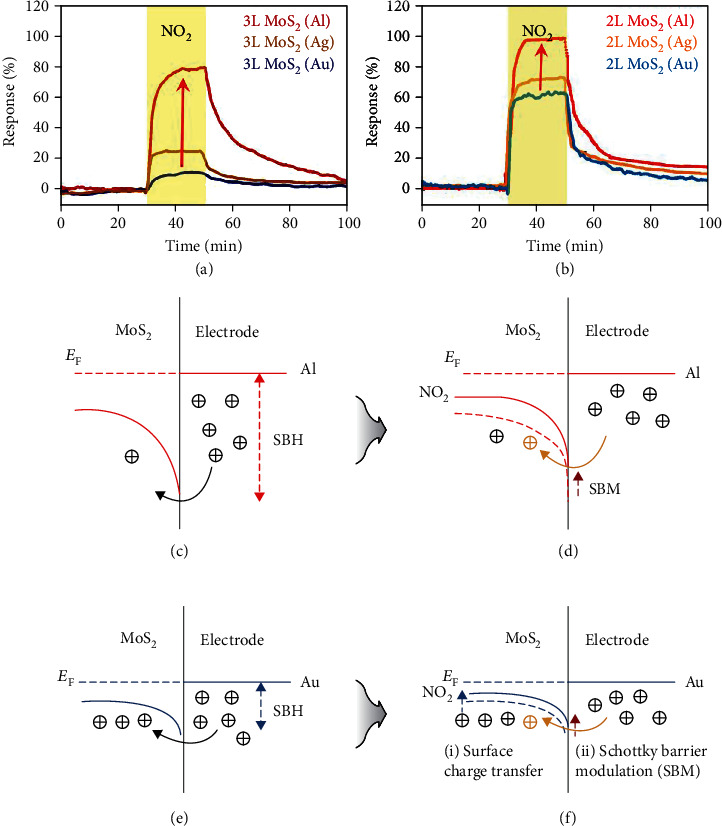
Sensing characteristics of NO_2_ for (a) 3-layer (3L) MoS_2_ and (b) 2L MoS_2_ with Al, Ag, and Au electrodes. Band diagram of the Al/MoS_2_ gas sensor (c) before and (d) after NO_2_ exposure. Band diagram of the Au/MoS_2_ gas sensor (e) before and (f) after NO_2_ exposure [81], copyright 2019 IEEE.

Here, *q* is the electron charge, *n* is the ideality factor, *k* is the Boltzman constant, *T* is the absolute temperature, *S* is the contact area, *A* is the Richardson constant, and *Ø*_b_ is the Schottky barrier height. The above two equations indicate that the current varies exponentially with the Schottky barrier height. Therefore, the charge transfer upon gas adsorption, which can change the Fermi level of the semiconductor and in turn vary the Schottky barrier height [80], can lead to a large current response of the sensor. As an example, Kim et al. [81] fabricated p-type MoS_2_-based gas sensors with three kinds of metal electrodes, i.e., Au, Ag, and Al. The sensors with the Al electrode exhibited the best sensing response toward NO_2_ (Figures 5(a) and 5(b)). This was attributed to the lower work function of Al (4.06 eV) as compared with Au (5.1 eV) and Ag (4.26 eV), thus the higher Schottky barrier height when in contact with MoS_2_ (Figures 5(c) and 5(e)). After the NO_2_ adsorption, the amplitude of the change of the Schottky barrier height in the Al-MoS_2_-based gas sensor was higher than the others (Figures 5(d) and 5(f)), leading to the highest sensing response.

As for the Ohmic contact, although it is characterized by a linear *I*-*V* curve with a relatively low contact resistance as compared to the Schottky contact [82], the change of its resistance, on the contrary, is less affected by gas adsorption.

*(2) Layer Number*. The electronic properties of 2D materials, such as band levels and carrier mobilities, are very much dependent on their thickness or layer number [29, 55, 83, 84]. This, as expected, can lead to the layer number-dependent gas sensing behavior.

It is generally accepted that thinner nanosheets can provide a larger surface-to-volume ratio for gas adsorption, and the enlarged bandgap and varied band positions may help tune the energy barriers for charge transfer with gas molecules. However, ultrathin 2D materials are prone to environmental perturbations. For example, single- or few-layer MoS_2_ showed lower mobilities than multilayer or bulk MoS_2_, which is likely caused by the Coulomb potential built up by charges trapped in the substrate (e.g., Si/SiO_2_) [85, 86]. In addition, a reduced carrier concentration in thinner nanosheets may also pose an adverse effect on its sensing response [29, 85, 87]. For example, Li and coworkers found that a single-layer MoS_2_ FET sensor showed an unstable and lower response toward NO as compared to double- to four-layer MoS_2_ [88]. Similarly, Late et al. [55] observed poor sensing performance in a double-layer MoS_2_-based FET gas sensor as compared to a five-layer MoS_2_ FET.

Increasing the layer number of 2D materials may provide a reduced gas binding energy at interlayer adsorption sites, bringing benefit to gas sensing. Through first-principle calculations, Qin et al. [61] suggested that the interlayer adsorption sites for NH_3_ in few-layer or bulk WS_2_ have higher binding energy (-0.356 eV) than the surface adsorption sites on a monolayer WS_2_ (-0.179 eV), and the corresponding net charge transferred is 0.038 and 0.006 e, respectively. Besides, the recovery time and sensing response of the thicker nanosheets were found to be both larger than the thinner ones. It is however worth noting that too high a binding energy, such as that involved in chemisorption, may not be desirable for gas sensing due to the difficulty in gas desorption.

On the basis of the above discussion, it can be inferred that although tuning MC thicknesses can change a number of their properties such as charge carrier mobility, carrier density, band levels, and specific affinity toward gas adsorption, the dominating factor or factors that control the gas sensing performance require systematic investigations and consideration case by case.

**Figure 6:**
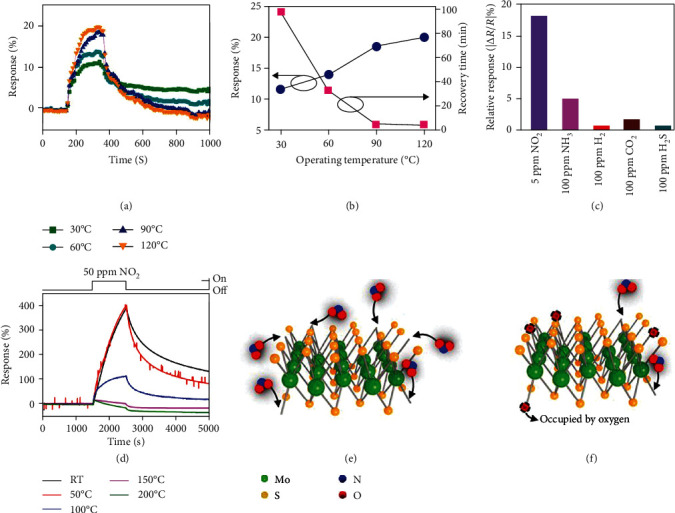
(a) Typical sensing response curves of the MoSe_2_ nanosheet-based sensor toward 20 ppm ethanol at different temperatures. (b) Response and recovery time of the MoSe_2_ nanosheet sensor as a function of operating temperatures. (c) Relative response of various gases at the optimum temperature (60°C) of the sensor [57], copyright 2019 Elsevier Ltd. (d) Transient response of the SiO_2_ nanorods encapsulated by MoS_2_ to 50 ppm NO_2_ at different operating temperatures. Schematic illustration of the reaction mechanism at (e) room temperature and (f) high operating temperatures [60], copyright 2018 American Chemical Society.

*(3) Working Temperature*. Temperature is an important parameter affecting gas sensing performance, especially for electrical sensors [89]. Raising the temperature can increase the carrier concentration of a semiconducting sensing material, promote surface reactions, and result in a higher response; besides, higher temperatures can also enhance the kinetics of the gas adsorption and desorption process, simultaneously influencing the profile of the response curve [22, 90]. An early investigation of the temperature effect by Zhang et al. [57] suggests that increasing the temperature would lower the energy barrier for ethanol adsorption on MoSe_2_, resulting in the enhanced charge transfer and higher response (Figures 6(a) and 6(b)).

However, when a sensor operates in the air, interference from water and other gases would complicate the temperature effect. Conventional metal oxide-based gas sensors require elevated working temperatures (typically >150°C), because target gas molecules interact strongly with preadsorbed high-temperature oxygen species, and the conductance of a metal oxide improves at higher temperatures [91]. A similar principle can be applied to MC-based sensors. Kumar et al. [62] reported that a NO_2_ sensor based on a MoS_2_ nanowire network showed a higher response at 60°C than at room temperature. Apart from the conductivity improvement, raising the temperature to 60°C could induce desorption of oxygen and water molecules originally capped on the MoS_2_ surface, thus providing more room for NO_2_-MoS_2_ interaction. As a result, a good selectivity at 60°C was also achieved with this sensor (Figure 6(c)). At further elevated temperatures, for example, 120°C, a sharp decrease of the sensing response was observed, probably because the desorption of NO_2_ surpassed its adsorption as the interaction between NO_2_ and MoS_2_ is an exothermic process. Similarly, Shim et al. also observed a reduced sensing performance of a MoS_2_-based NO_2_ sensor when the temperature was increased from 50 to 200°C (Figure 6(d)) [60]. They suggested that high-temperature oxygen species, i.e., O_2_^−^, O^−^, and O^2−^, might emerge at elevated temperatures (e.g., 150°C) and occupy the surface active sites of MoS_2_ (Figures 6(e) and 6(f)). From the above two examples, it can be seen that at room temperature to slightly elevated temperatures (e.g., 60°C), a target gas like NO_2_ will compete with O_2_ and H_2_O in the air to be absorbed on the sensing material; at further elevated temperatures, most O_2_ and H_2_O molecules will desorb from the sensor surface and instead oxygen anions will form and compete with NO_2_ for adsorption sites.

It is worth noting that, for the detection of electron-accepting gases such as NO_2_ or NO, common interference gases like O_2_ and H_2_O in the air may affect the selectivity of the sensor due to their competition with the target gas for absorption sites on the sensor surface. Therefore, a careful choice of the working temperature may help eliminate the influence from O_2_ and H_2_O and achieve better selectivity. On the other hand, for the detection of electron-donating gases like NH_3_ or acetone, the O_2_^−^, O^−^, and O^2−^ species present at elevated temperatures are beneficial for the improvement of the sensor selectivity and sensitivity owing to the interaction between these oxygen species and the electron-donating gases.

**Figure 7:**
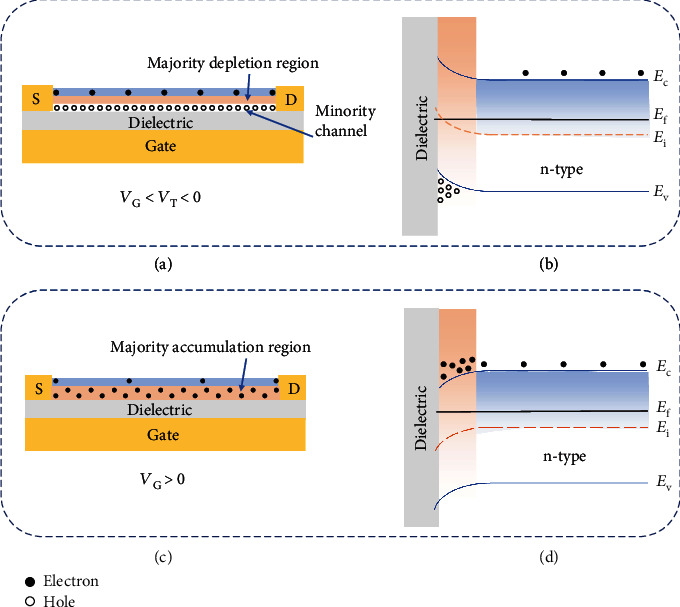
Schematic illustration of two modes of applying a gate bias to tune the performance of an FET gas sensor: (a) formation of the minority carrier channel at *V*_G_ < *V*_T_ < 0, and the (b) corresponding energy band level diagram; (c) formation of the majority carrier accumulation region at *V*_G_ > 0, and the (d) corresponding energy band level diagram. *E*_c_, *E*_f_, *E*_i_, and *E*_v_ denote the conduction band, Fermi level, intrinsic Fermi level, and valence band, respectively.

*(4) Application of the Gate Bias for FET Sensors*. Most FET gas sensors adopt the bottom-gated configuration, where the semiconducting channel is directly exposed to the target gas. Taking the n-type channel as an example, applying a negative gate bias (*V*_G_ < *V*_T_ < 0, where *V*_G_ is the gate voltage and *V*_T_ is the threshold voltage) can increase the hole concentration and form the minority carrier channel between the drain and the source electrode (Figures 7(a) and 7(b)) [89, 92, 93]. In the case of *V*_G_ > 0 (Figures 7(c) and 7(d)), the bias can induce an electron accumulation region. The bias-induced formation of either a charge depletion or an accumulation region can assist in modifying the gas sensing behavior of the device. An example to illustrate this approach would be the n-type MoS_2_ nanosheet-based FET sensor fabricated by Late et al. [55]. When a positive gate bias is applied to the sensor, additional electrons are accumulated at the contact interface between the MoS_2_ and the dielectric gate material. Upon exposure to oxidizing gases like NO_2_, electrons from the accumulation region interact with and are transferred to NO_2_, leading to the enhanced sensing performance; oppositely, when the sensor is exposed to reducing gases like NH_3_, the accumulated electrons would repel electrons from NH_3_ molecules and thus in turn decrease the sensing response.

#### 2.1.2. Sensing Involving Surface Reactions

**Figure 8:**
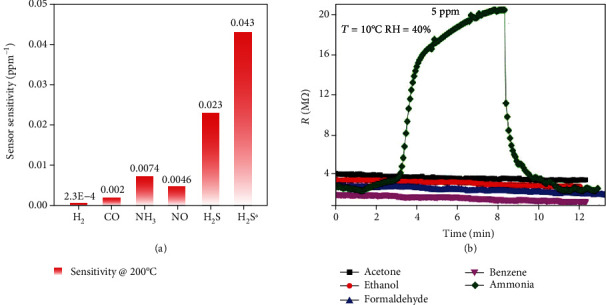
(a) Sensitivity of the five different sensors, displaying high selectivity toward H_2_S (0.023 ppm^–1^ at 1 ppm). The data point labeled with an asterisk denotes the sensitivity (0.043 ppm^–1^) measured at 20 ppb H_2_S [68], copyright 2018 Springer Nature. (b) The selectivity of the WS_2_ nanoflake-based sensor to different gases at RH = 40% [67], copyright 2017 Elsevier Ltd.

Gas sensing with conventional metal oxide-based gas sensors usually involves surface reactions between preabsorbed oxygen species (O^2−^, O^−^, and O_2_^−^) and target gas molecules at elevated temperatures [22, 94–98]. Likewise, chemical reactions on MC surfaces can also take place at elevated temperatures, leading to improved sensing performance. For example, Asres et al. reported that a WS_2_-based gas sensor exhibited excellent sensitivity (0.043 ppm^–1^) and high selectivity (Figure 8(a)) toward H_2_S at 200°C [68]. They suggested that under ambient conditions, the O_2_ in the air can substitute some of the S atoms in WS_2_ to form WS_2-*x*_O*_x_*. When exposed to H_2_S at elevated temperatures, part of the O atoms in WS_2-*x*_O*_x_* could be replaced by S from H_2_S to yield WS_2-*y*_O*_y_* until a new equilibrium is reached.

In another interesting work, Li et al. demonstrated that the sensing performance of WS_2_ nanoflakes toward NH_3_ could be improved at higher humidity due to the proposed hydroxylation reaction as shown below [67]:

(3) $$\begin{array}{c} {\text{SO}}_{4}^{2-}\;\left(\text{W}{\text{S}}_{2}\text{ surface}\right)+{\text{H}}_{2}\text{O}⟷{\text{H}}^{+}+{\text{SO}}_{4}^{2-}+{\text{OH}}^{-} \end{array}$$

The increased surface acidity in humid conditions could help attract more basic NH_3_ molecules to donate electrons. Besides, higher humidity may enable more NH_3_ molecules bound to the WS_2_ surface via the “solvation” effect according to the equation below:

(4) $$\begin{array}{c} {\text{NH}}_{3}+{\text{H}}_{2}\text{O}⟷{\text{NH}}_{4}^{+}+\text{O}{\text{H}}^{-} \end{array}$$

The resulting NH_4_^+^ ions would further react with the adsorbed oxygen ions to donate electrons to WS_2_:

(5) $$\begin{array}{c} {\text{NH}}_{4}^{+}+{\text{O}}_{2}^{-}⟶\text{NO}+{\text{H}}_{2}\text{O}+{\text{e}}^{-} \end{array}$$

Because of the reaction-assisted sensing pathway, this sensor exhibits an unrivaled selectivity toward NH_3_ against other electron-donating gases such as acetone and ethanol (Figure 8(b)).

It can be inferred from the above examples that surface reactions with target gases on MC-based sensing materials mainly involve water vapor- and oxygen-related species (i.e., O_2_, O_2_^−^, O^−^, and O^2−^) in the air. Although these reactions may enhance the sensing response and selectivity, they may also bring problems of long response time, slow desorption, and poor reusability.

#### 2.1.3. Sensing via Proton Conduction

**Figure 9:**
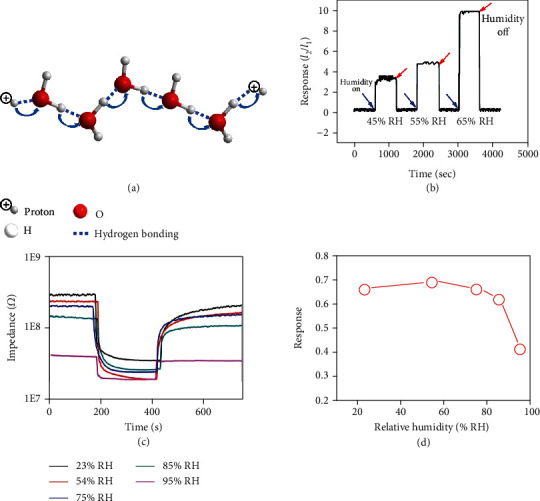
(a) Schematic illustration of the Grotthuss mechanism. (b) Response of sonication-exfoliated MoS_2_ nanosheets for three different humidity levels [65], copyright 2016 The Royal Society of Chemistry. (c) The transient response-recovery curves of a WS_2_-based impedance sensor to 5 ppm NO_2_ measured at 100 Hz at 25°C in different humidity levels. (d) The gas sensor response to 5 ppm NO_2_ as a function of the relative humidity [70], copyright 2018 Elsevier Ltd.

One of the most accepted proton conduction mechanisms, the Grotthuss mechanism or the hopping mechanism, was proposed in 1806 by Theodor von Grotthuss and named after him [99]. This process exists in all liquid water, in which protons are tunneled from one water molecule to an adjacent one through hydrogen bonding (Figure 9(a)) [66]. The proton conduction based on the Grotthuss mechanism therefore has also been proposed as a plausible mechanism to explain many humidity sensing phenomena in addition to electron/hole transfer [66, 100, 101]. For example, MoS_2_ nanosheets were used for humidity sensing by Burman et al. [65]. They suggested that at relatively low humidity, water molecules chemisorbed on MoS_2_ surfaces are dissociated into hydroxyl ions (OH^−^) and protons (H^+^), and the OH^−^ ions were preferably adsorbed at the S-vacancy sites (Equation (6)) [102]. Because the formed water layer is not continuous at this stage to support the effective proton conduction, the main sensing mechanism is still based on charge transfer from water to n-type MoS_2_. As the relative humidity increases, more water molecules are physisorbed on top of the chemisorbed water layer to form a second layer and dissociated into hydronium groups (H_3_O^+^) and hydroxyl ions (OH^−^) (Equation (7)). Then, proton conduction via hopping can take place in both water layers, leading to a sudden rise of the sensing response (Figure 9(b)).

(6) $$\begin{array}{c} {\text{H}}_{2}\text{O}⟷{\text{H}}^{+}+{\text{OH}}^{-}\;\left(\text{for lower humidity levels}\right) \end{array}$$

(7) $$\begin{array}{c} 2{\text{H}}_{2}\text{O}⟷{\text{H}}_{3}{\text{O}}^{+}+{\text{OH}}^{-}\;\left(\text{for higher humidity levels}\right) \end{array}$$

Since MCs are generally sensitive to humidity to various extent, humidity becomes an unneglectable influencing factor when MCs are used for gas sensing in the air. As reported by Xu et al. [70], their WS_2_-based impedance NO_2_ sensor showed a reduced baseline with increasing humidity. This is because H_2_O molecules adsorbed on WS_2_ would be dissociated into H^+^ or H_3_O^+^ ions, which, under an electrostatic field, could transport via the hopping mechanism (Figure 9(c)). The baseline change led to a reduction in the sensing response (Figure 9(d)). This negative effect of humidity can be reduced via approaches such as noble metal decoration, coating with a hydrophobic film, and construction of p-n junctions [22, 103, 104].

#### 2.1.4. Sensing via Change of the Refractive Index

As mentioned in the introduction (Figures 1(d) and 1(e)), fiber-based optical gas sensors, which are based on gas adsorption-induced change of the refractive index, have been combined with 2D materials for improved selectivity. For example, Sangeetha and Madhan [105] substituted a portion of the cladding with the graphene-MoS_2_ nanoparticle composite, whose refractive index changed upon gas adsorption, leading to a change in the evanescent field, further influencing the light intensity transmitted through the waveguide. This sensor achieved good sensitivity (61%), rapid response (22 s), short recovery time (35 s), and appreciable selectivity, as well as good stability toward 500 ppm NO_2_. It is important to note that although the sensitivity of this type of sensor is not comparable with electrical sensors which can detect sub-ppm level NO_2_, they are suitable for sensing at long distances, especially in situations involving dangerous environments [106].

Compared to the measurement of light intensity, monitoring the shift of the light wavelength may provide better selectivity and precision. Taking the advantage of PC-based optical sensors, Zhao et al. [107] incorporated MoS_2_ with the SiO_2_-based PC cavity slab in a fiber optic sensor for methanol detection. The refractive index of MoS_2_ varies when methanol molecules are adsorbed on its surface, which shifts the resonance wavelength of the PC. Different from electrical sensors whose selectivity mainly comes from the ability of gases to donate or withdraw electrons, the selectivity of this type of optical sensor mainly depends on the polarity of the gases in addition to the strength of the interaction between the gas and the MoS_2_. Although the sensor responds not only to methanol but also to acetone and ether, it shows the highest response to methanol, which is important since selective methanol detection has been difficult to be realized with electrical sensors. More importantly, this sensor showed an ultrafast response (300 ms) and could enable the real-time monitoring of the light spectrum when coupled with IR adsorption spectroscopy or Raman scattering spectroscopy [108, 109].

### 2.2. Approaches to Improve Sensing Performance

**Figure 10:**
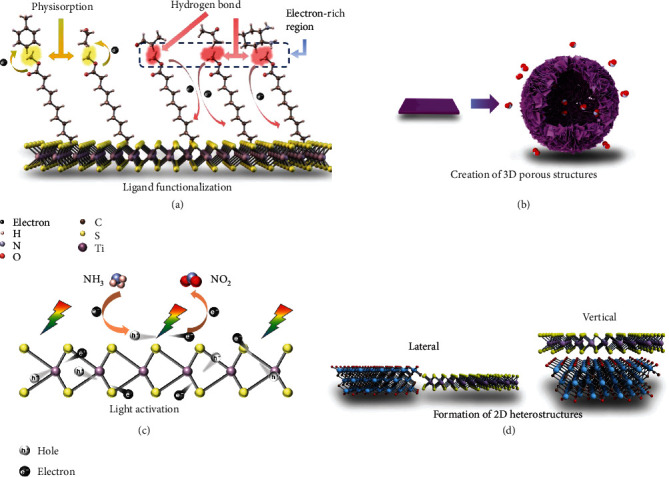
Schematic illustration of strategies for improving the gas sensing performance: (a) ligand functionalization, (b) creation of 3D porous structures, (c) light activation, and (d) formation of 2D heterostructures.

To date, various approaches have been explored to enhance the performance of gas sensors based on 2D materials, such as ligand functionalization, creation of 3D porous structures, light activation, and formation of 2D heterostructures (Figure 10), which are introduced in the following context.

#### 2.2.1. Ligand Functionalization

**Figure 11:**
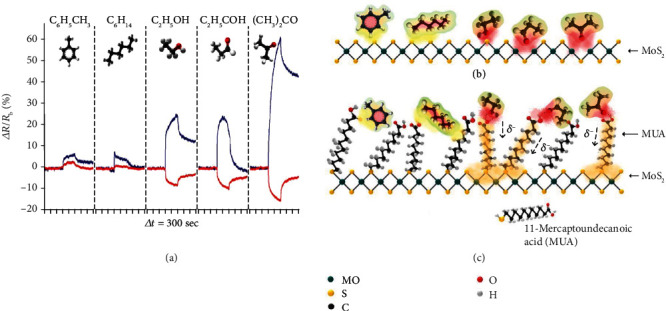
(a) Sensor responses of primitive (blue curves) and MUA-conjugated (red curves) MoS_2_ sensors for target VOCs. Schematic illustration of the interaction between the VOC molecules and the surface of (b) primitive MoS_2_ and (c) MUA-conjugated MoS_2_ [112], copyright 2015 American Chemical Society.

The functionalization of MCs with organic molecules can modulate their surface chemistry, surface charge states, and electronic structures [45, 110, 111], which in turn modifies their gas sensing behaviors. For example, Kim et al. [112] functionalized the MoS_2_ surface with mercaptoundecanoic acid (MUA) and compared its gas sensing performance with that of primitive MoS_2_. The primitive MoS_2_ showed an increased resistance toward five different gases, including toluene, hexane, ethanol, propionaldehyde, and acetone, regardless of their redox nature (Figure 11(a)). Therefore, the gas dipole-induced charge scattering was proposed to be the dominant mechanism behind the conductance reduction (Figure 11(b)). In sharp contrast, the MUA-conjugated MoS_2_ showed decreased resistance upon exposure to oxygen-functionalized gases including ethanol, propionaldehyde, and acetone. This is because the carboxyl groups of MUA molecules could interact with the oxygen-containing functional groups in these gas molecules via the formation of hydrogen bonds to promote the electron transfer toward MoS_2_ through the saturated alkyl chains of MUA (Figure 11(c)). It is interesting to note that by surface modification, the sign of the sensing response to a specific gas can be inverted, providing another means for selectivity enhancement.

#### 2.2.2. Creation of Porous 3D Assemblies of MCs

**Figure 12:**
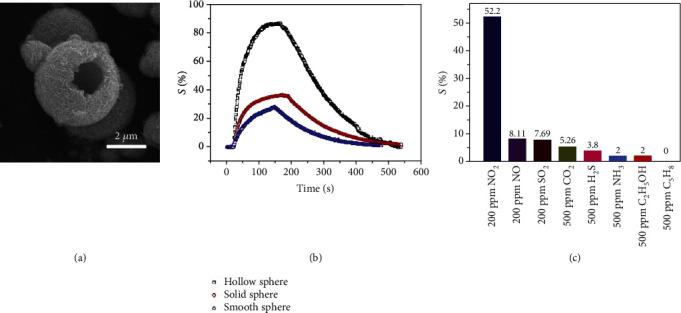
(a) SEM image and (b) sensing response of hierarchical hollow MoS_2_ spheres in comparison with solid and smooth spheres. (c) Responses of hollow sphere-based sensors to 8 different gases (500 ppm H_2_S, NH_3_, C_2_H_5_OH, C_5_H_8_, and CO_2_; 200 ppm NO_2_, SO_2_, and NO) at the optimal temperature [116], copyright 2019 Elsevier Ltd.

The construction of porous 3D assemblies of MCs is able to maximize their exposed surfaces for gas-solid interactions and enables fast gas diffusion and recovery kinetics [62, 113–115]. This was recently demonstrated by Li et al. [116], who showed that hierarchical hollow MoS_2_ microspheres exhibited a higher specific surface area and improved gas permeability, thus leading to an enhanced gas sensing performance toward NO_2_ as compared to solid spheres or smooth spheres (Figures 12(a) and 12(b)). It is noteworthy that the selectivity of the sensor is also good (Figure 12(c)), which was attributed to not only the specific interaction between NO_2_ and MoS_2_ but also the size-selective penetration of different gases through the pores of the hierarchical hollow MoS_2_ microspheres. In another interesting work by Asres et al. [68], 1D nanowires and 2D nanosheets of WS_2_ were combined together for H_2_S sensing. While the nanowires provided a continuous conductive network with percolated channels for gas diffusion, the thin 2D nanosheets provided large surface areas to adsorb gas molecules on both basal faces and edges.

#### 2.2.3. Light Activation

**Figure 13:**
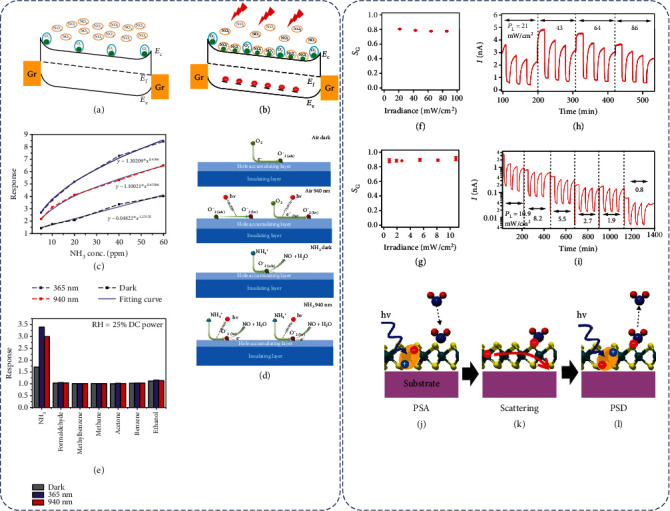
Band diagram of the Au/MoS_2_/Au sensor (a) in the dark and (b) under red light illumination [64], copyright 2019 American Chemical Society. (c) The correlation curve of the response of the WS_2_-based sensor under the light (365 nm, 940 nm) illumination and the dark. (d) Schematic illustration of the possible 940 nm light-enhanced gas sensing mechanism of WS_2_-based sensors to NH_3_ at low temperature. (e) Comparison of the response of the sensor under the light (365 nm, 940 nm) illumination driven by direct current power and the dark to several possible interferents such as formaldehyde, methylbenzene, methanol, acetone, benzene, and ethanol with 60 ppm each [117], copyright 2018 Elsevier Ltd. Irradiance dependences of the sensor response *S*_G_ to 100 ppb NO_2_ under light from (f) a solar simulator or (g) a blue LED. Changes in the drain current of a sensor exposed to cyclic exposure of NO_2_ (100 ppb) in the air under light illumination from (h) a solar simulator and (i) a blue LED with various irradiances (*P*_L_). Schematics of (j) photostimulated adsorption, (k) carrier scattering caused by adsorbed NO_2_ molecules, and (l) photostimulated desorption [126], copyright 2021 American Chemical Society.

Light illumination on semiconductors can enhance their sensing performance by inducing photogenerated charge carriers [117]. For example, Pham et al. [64] reported that an n-type MoS_2_-based optoelectronic NO_2_ sensor exhibited an improved sensing response under the red light illumination as compared to the dark. It was suggested that the oxygen ions originally absorbed on the surface of MoS_2_ could trap electrons to prevent them from interacting with NO_2_ (Figure 13(a)). The additional electrons generated by red light activation were not bound to the oxygen ions, and some of them were free to be transferred to NO_2_ molecules to raise the sensing response (Figure 13(b)).

Different excitation energies can have different effects on the sensing response. In a very systematic study, Gu et al. [117] found that both ultraviolet (UV) light (365 nm) and near-infrared (NIR) light (940 nm) can help improve the performance of an WS_2_-based NH_3_ sensor at 40°C (Figure 13(c)), but are based on different mechanisms. Under 940 nm light illumination, which is about the bandgap of WS_2_, the photogenerated electrons could interact with O_2_ to yield O_2_^−^_(hv)_ ions absorbed on WS_2_. These O_2_^−^_(hv)_ ions, as compared to the preabsorbed oxygen ions (O_2_^−^_(ads)_) under dark conditions, are more energetic to interact with the target NH_3_ gas molecules [117–122], thus resulting in the enhanced sensing performance. Under 365 nm illumination, on the other hand, the light energy was able to excite not only WS_2_ but also electrons localized in the highest occupied molecule orbit (HOMO) of NH_3_ from their ground state [117, 123–125]; these excited electrons in NH_3_ were more likely to be transferred to WS_2_, resulting in a larger resistance change (Figure 13(d)). Furthermore, because of this light activation of NH_3_ molecules, the sensor showed the best selectivity under 365 nm light excitation as compared to under 940 nm light or without light (Figure 13(e)).

A new sensing mechanism was recently reported by Tabata et al. [126]. They found that the common charge transfer mechanism cannot explain the sensing phenomenon they observed with a MoS_2_ monolayer-based NO_2_ sensor under light activation. As shown in Figures 13(f)–13(i), the response values are almost independent of the light irradiance (or power). If charge transfer is the main mechanism, at varied radiances, the amount of charge transferred at the same gas concentration should be the same, whereas the film current should vary with irradiance due to the different amounts of photoinduced charge carriers. Therefore, the sensor response should also vary with radiance, which contradicts the experimental observation. The authors thus proposed that the adsorbed NO_2_ molecules do not change the density of photoexcited carriers in MoS_2_; rather, they act as scattering centers to disrupt electron drifting in MoS_2_, which reduces the carrier mobility. In addition, the photostimulated adsorption and desorption could increase the response and recovery rate (Figures 13(j)–13(l)).

In addition, the light illumination is not only able to alter the carrier concentration of the sensing material but also able to modify the properties of certain target gas molecules. For example, Wu et al. [59] observed opposite sensing responses of a MoTe_2_-based acetone sensor with and without light illumination. This is because the UV activation of the acetyl group of acetone can induce its transformation from a weak reducing agent into a weak oxidizing agent. Such unique chemical characteristic of ketone molecules may enable their selective detection in a VOC mixture.

#### 2.2.4. Formation of MC-Based Heterostructures

Heterostructures that combine dissimilar 2D materials have shown interesting properties that differ from their individual 2D components [127, 128]. Based on the different spatial arrangements of the 2D constituents in a heterostructure, they can be classified as vertical or lateral heterostructures. The vertical heterostructures can be created via stacking different 2D crystals one above another, stabilized by the weak van der Waals force [129–132]. The lateral heterostructures are constructed from edge-connected 2D crystals, creating 1D interfaces [133]. In terms of the type of constituent 2D materials, on the other hand, 2D heterostructures can be classified as either metal-semiconductor heterostructures or semiconductor-semiconductor heterostructures. Based on such classification, in the following context, we introduce gas sensors whose performances are enhanced via the formation of heterostructures.

*(1) Metal-Semiconductor MC Heterostructures.* For many metal chalcogenides, their electronic properties, for example, whether they are metallic or semiconducting, are largely dependent on their compositions and crystal phases [134, 135]. Therefore, metal-semiconductor MC heterostructures can be designed and prepared via controllable synthesis and postsynthesis treatment. As mentioned in Section (4), a Schottky barrier or Ohmic contact can form at the interface between a metal and a semiconductor, depending on the semiconductor type and the relative position of their work functions.

**Figure 14:**
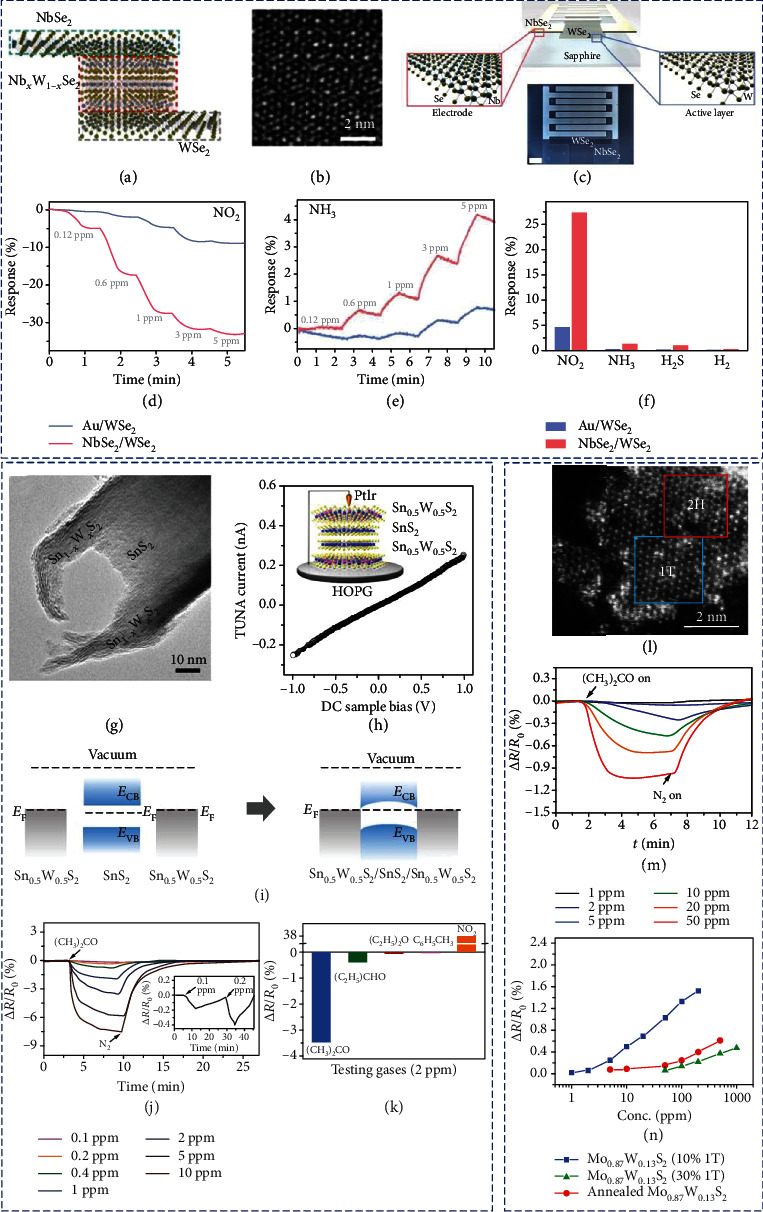
(a) Schematic of the cross-sectional crystal structure of the NbSe_2_ (metallic layer)-Nb*_x_*W_1-*x*_Se_2_ (transition layer)-WSe_2_ (semiconducting layer) heterojunction. (b) Atomic resolution annular dark-field (ADF) scanning transmission electron microscopy (ADF-STEM) image of the Nb*_x_*W_1-*x*_Se_2_ transition layer. (c) Schematic image of the NbSe_2_/WSe_2_ gas sensing device and crystal structure of the metallic NbSe_2_ (left red box) and semiconducting WSe_2_ (right blue box). Transient resistance responses to (d) NO_2_ and (e) NH_3_ analyte gases for both NbSe_2_/WSe_2_ and Au/WSe_2_. (f) Gas responses under various gases (NO_2_, NH_3_, H_2_S, and H_2_) at concentrations of 1 ppm for both devices [136], copyright 2016 American Chemical Society. (g) Side-view TEM image of the Sn_1–*x*_W*_x_*S_2_/SnS_2_ heterostructure. (h) *I*-*V* curves measured with tunneling atomic force microscopy (TUNA) for a Sn_0.5_W_0.5_S_2_/SnS_2_ heterostructure, under a constant force and an applied bias voltage that was linearly ramped down. (i) Schematic band alignment diagram for Sn_0.5_W_0.5_S_2_/SnS_2_ and SnS_2_ before and after contact. *E*_F_, *E*_CB_, and *E*_VB_ denote the Fermi level, conduction band, and valence band, respectively. (j) Response-recovery curves of a typical chemiresistive sensor fabricated from Sn_0.5_W_0.5_S_2_/SnS_2_ heterostructures in response to acetone gas with increasing concentrations. Inset: zoomed-in response of the sensor toward 0.1 and 0.2 ppm acetone. (k) Comparison of the responses of the sensor toward different gases, including acetone, diethyl ether, propanal, toluene, and NO_2_ [137], copyright 2018 Nature Publishing Group. (l) TEM image of a thin Mo_1-*x*_W*_x_*S_2_ layer. (m) Normalized resistance changes of a typical chemoreceptive sensor fabricated from Mo_0.87_W_0.13_S_2_ (∼10% 1T) in response to acetone gas with increasing concentrations. (n) Normalized change of resistance of different sensors at various acetone concentrations [138], copyright 2017 The Royal Society of Chemistry.

Cho et al. prepared vertical metallic NbSe_2_/semiconducting WSe_2_ heterostructures with a Nb*_x_*W_1-*x*_Se_2_ transition layer at the heterojunction (Figures 14(a)–14(c)) by selenization of WO_3_ and Nb_2_O_5_ films sequentially deposited on a sapphire substrate via the CVD method [136]. Note that the original Schottky barrier height between the p-type WSe_2_ sensing layer and the Au electrode was 94 meV. This was considerably reduced to 25 meV when WSe_2_ was made in contact with the metallic NbSe_2_ via the Nb*_x_*W_1-*x*_Se_2_ transition layer. The much-reduced barrier height for charge transfer across the electrode-semiconductor interface resulted in a rise in responses toward both NO_2_ and NH_3_ (Figures 14(d) and 14(e)). The enhancement in sensing of NO_2_ is more profound than that in sensing of NH_3_, resulting in high selectivity toward NO_2_ (Figure 14(f)).

Metallic 2D materials can act not only as the electrode but also as the active sensing material when interfacing with semiconducting 2D materials. Wang and coworkers epitaxially deposited NH_4_^+^-intercalated Sn_0.5_W_0.5_S_2_ nanosheets on the top and bottom surfaces of n-type SnS_2_ nanoplates to form vertical metal-semiconductor heterostructures with the Ohmic-type interface (Figures 14(g)–14(i)) [137]. The acetone sensing film fabricated from such sandwiched nanoheterostructures exhibited a much-reduced film resistance. As a result, a 35 times lowered background noise and a higher signal-to-noise ratio were achieved. Besides, the formation of Sn_0.5_W_0.5_S_2_ alloy led to increased adsorption energy toward acetone, realizing selective acetone sensing at the 100 ppb level (Figure 14(j)). In addition, compared to other gases like diethyl ether and propanal, acetone has a stronger ability to donate electrons, leading to an appreciable selectivity (Figure 14(k)).

Crystal phase heterostructures of MCs which are constructed from chemically homogeneous but structurally different domains have also shown interesting electronic properties [139]. The use of such phase heterostructures in gas sensing was demonstrated by Yang et al. [138], who prepared alloyed Mo_1-*x*_W*_x_*S_2_ nanosheets with tunable 1T/2H phase ratios via a one-pot hydrothermal process. Due to the intercalation of NH_4_^+^ ions released by the reaction reagent, semiconducting 2H Mo_1-*x*_W*_x_*S_2_ was partially in situ converted to metallic 1T structures, forming phase heterostructures made of randomly distributed domains of 1T and 2H phases (Figure 14(l)). The produced Mo_0.87_W_0.13_S_2_ nanosheets with a 1T concentration of ∼10% showed the best gas sensing performance toward acetone, which was attributed to an optimized combination of the conductive 1T domains and the metal-semiconductor heterointerfaces between the 1T and 2H phases (Figures 14(m) and 14(n))

*(2) Semiconductor-Semiconductor MC-Based Heterostructures.* When an n-type semiconductor and a p-type semiconductor are brought into contact, the different carrier concentrations at the interface induce carrier diffusion, and a charge depletion layer with a built-in potential is formed, which controls the charge flow at the heterointerface. Given the exponential relationship between the current and the energy barrier at the heterojunction, gas-induced modulation of the barrier height can lead to a sharp change of the channel current, i.e., a large sensing response [75, 78, 79, 140]. For example, Feng et al. prepared a NO_2_ sensor based on BP/MoSe_2_ vertical heterostructures through stacking p-type BP on n-type MoSe_2_ flakes exfoliated from their respective bulk crystals [74]. The gas sensing performance of the BP/MoSe_2_ sensor toward NO_2_ was significantly improved, exhibiting 4.4 and 46 times higher response toward 200 ppb NO_2_ as compared to that of a pure BP sensor or MoSe_2_ FET sensor, respectively. The authors simulated the band diagram of the BP/MoSe_2_ heterojunction before and after NO_2_ adsorption at equilibrium conditions. The result indicates a rise of the total built-in potential at the junction, i.e., from 0.30 to 0.36 eV, leading to a higher barrier for electron transport and a much-reduced channel conductance.

## 3. MXenes

**Figure 15:**
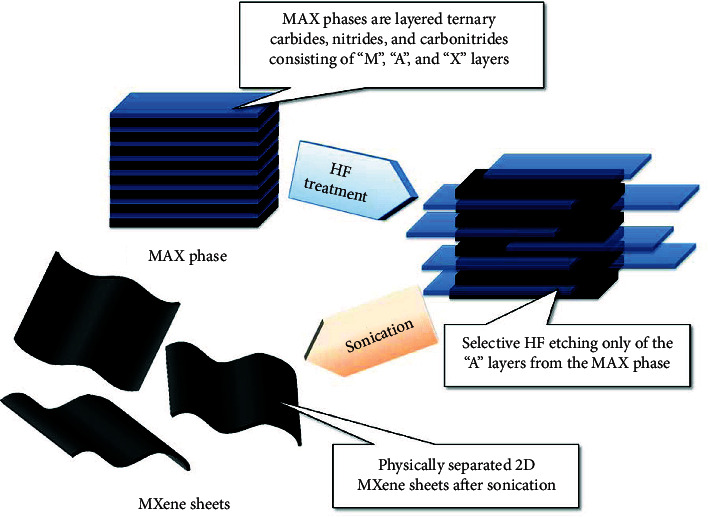
Schematic for the exfoliation process of MAX phases and formation of MXenes [153], copyright 2012 American Chemical Society.

MXenes are an emerging family of 2D materials, including layered metal carbides, carbonitrides, and nitrides, with a general formula of M_*n*+1_X*_n_*, where M is a transition metal (such as Ti, Nb, and Mo) and X is carbon and/or nitrogen [142–144]. MXenes are normally prepared from layered ternary metal carbides/nitrides, also called MAXs, through selective etching away the “A” layer (group IIIA or IVA elements, e.g., Al, Si, and Ge) (Figure 15) [145]. Till now, over 20 types of MXenes have been discovered, experimentally prepared, and explored in energy storage [146], desalination [147], electromagnetic interference shielding [148], catalysis [149], sensing [150], and other fields [151].

Unlike chemically modified graphene, whose surface functionalities and hydrophilicity are normally achieved at the expense of their electrical conductivity, MXenes exhibit excellent electrical conductivity and possess hydrophilic surfaces with functional groups [145, 148, 152]. These features make them appealing candidates in electrical sensing devices, where good electrical conductivity for low background noise and surface functional groups for interacting with gas molecules are both essential requirements.

### 3.1. Sensing Mechanisms

Similar to MCs, charge transfer upon gas adsorption has been proposed as a possible mechanism for MXene-based gas sensors [1, 154]; however, some contradictory phenomena have been observed. For example, Kim et al. [152] found that a Ti_3_C_2_T*_x_*-based sensor showed increased channel resistance regardless of whether the target gas is donating or withdrawing electrons. This is in sharp contrast to the dopant type-dependent gas sensing behavior observed in most semiconducting sensing materials. One possible explanation is that most MXenes are narrow-bandgap semiconductors or metal-like, and thus, charge transfer from the target gas could not cause much change in their conductivity [152]. On the contrary, the gas adsorption-induced charge scattering, which reduces the carrier mobility of MXene, might become the dominant cause of the resistance increase [155].

Whether a MXene-based gas sensor is working based on the charge transfer, carrier scattering, or both, its sensing behavior is dependent on factors such as its composition, surface chemistry, and dopant type/concentration, as well as working temperature (Table 2) [155–158].

#### 3.1.1. Effect of Terminal Groups

Most MXenes can be prepared by HF etching or fluoride-based salt etching [145, 151, 159]. The etching process is sometimes accompanied by the ion intercalation into the interlayer spaces, for example, with large solvent molecules such as dimethyl sulfoxide (DMSO) under sonication [160]. Alternatively, the etching and delamination process can be combined into one step, especially when fluoride-based salts are used as the etchants [142, 161]. The processing conditions such as the type and concentration of etchant, the etching period and temperature, and the subsequent sonication time and temperature [151] all have influences on the size/thickness, crystallinity, and surface chemistry of the resulting MXene nanosheets/nanoflakes [142].

As-exfoliated MXenes generally have mixed terminal groups (-OH, -O, and -F) [162, 163], and the ratio between them is dependent on the exfoliation process and the subsequent treatment. For example, as-prepared MXene nanosheets can be rinsed with or stored in water to obtain a higher -OH ratio [164]; and a higher concentration of -O can be achieved by etching with fluoride-based salt rather than HF [162].

The electronic transport properties of MXenes could be influenced considerably by their surface terminal groups [165, 166]. Theoretical works have predicted that many MXenes are intrinsically metallic without surface groups (e.g., -F, -OH, or -O) [167, 168]. The surface functional groups may turn their intrinsic metallic nature into semiconducting with calculated bandgaps varying between 0.25 and 2.0 eV [154, 169]. Lee et al. [27] identified that as-prepared Ti_3_C_2_T*_x_* nanosheets were p-type semiconductors, and NH_3_ molecules could be absorbed preferentially on their surface defects or functional groups such as -O and -OH to donate electrons and increase the film resistance.

Among the -O, -OH, and -F terminal groups on Ti_3_C_2_T*_x_*, the -F group, as theoretically predicted, is likely to induce the largest electron transmission and thus the highest current at a given bias [166]. Therefore, controlling the relative -F concentration is able to effectively modulate the electrical conductivity of the MXene which is an important parameter affecting the gas sensing performance [150]. The -OH terminal group, on the other hand, could provide partially occupied nearly free electron (NFE) states in MXenes. The NFE states are located near the Fermi level and therefore can provide the hole and electron channels under low bias voltages. Examples of MXenes that exhibit the NFE states include Ti_2_C(OH)_2_, Zr_2_C(OH)_2_, Zr_2_N(OH)_2_, Hf_2_N(OH)_2_, Nb_2_C(OH)_2_, and Ta_2_C(OH)_2_ [169–171]. Importantly, the NFE states are sensitive to environmental perturbations; for example, they might be diminished upon adsorption of gases like O_2_, H_2_, and CO [169, 171]. Thus, MXenes with -OH terminal groups are suitable for gas sensors.

In most cases, -O, -OH, and -F terminal groups coexist in MXenes, and studies have found that different terminal ratios may lead to different affinities toward certain gases. By using the first-principle simulation, Hajian et al. [172] compared the gas adsorption behaviors of Ti_3_C_2_(OH)_0.44_F_0.88_O_0.66_ and Ti_3_C_2_(OH)_0.66_F_0.22_O_1.11_ toward NH_3_, CO_2_, NO, H_2_S, and SO_2_. Both MXenes showed stronger and specific interaction with NH_3_ than with other gases; the Ti_3_C_2_(OH)_0.66_F_0.22_O_1.11_ with a higher -O/-F termination ratio was found to be slightly more sensitive toward NH_3_, as indicated by a more negative adsorption energy (-0.49 eV) and a larger amount of charge transfer (0.099 e) as compared with Ti_3_C_2_(OH)_0.44_F_0.88_O_0.66_ (-0.36 eV and 0.098 e, respectively).

#### 3.1.2. Doping

The specific affinity of MXenes for a target gas can be controlled by elemental doping. For example, Mn-doped Sc_2_CO_2_ showed a much higher adsorption energy of -0.85 eV for CO as compared with pristine MXene (-0.14 eV), along with a 10 times increased charge transfer, i.e., from 0.017 e to 0.199 e, per CO molecule [173].

### 3.2. Modification of Sensing Properties

#### 3.2.1. Intercalation

**Figure 16:**
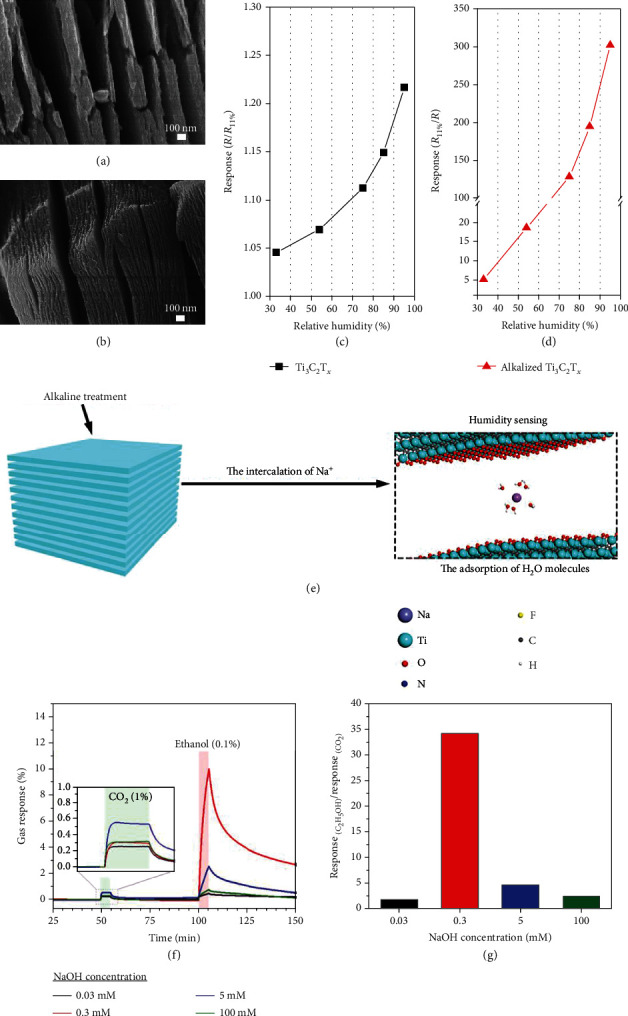
SEM images of (a) Ti_3_C_2_T*_x_* and (b) alkalized Ti_3_C_2_T*_x_*. Gas sensing performance of NaOH-treated Ti_3_C_2_T*_x_* sensors at room temperature. Response of the devices based on (c) Ti_3_C_2_T*_x_* and (d) alkalized Ti_3_C_2_T*_x_* to different relative humidity levels. (e) Schematic diagram of the adsorption of H_2_O molecules on the surface of alkalized Ti_3_C_2_T*_x_* [174], copyright 2019 American Chemical Society. (f) Real-time gas response behavior of Ti_3_C_2_T*_x_* sensors fabricated with various concentrations of NaOH (0.03, 0.3, 5, and 100 mM) upon exposure to 1% CO_2_ and 0.1% ethanol. The inset in (f) shows the magnified gas response of CO_2_. (g) Ethanol selectivity of gas sensors calculated by dividing the response toward ethanol over the response toward CO_2_ [176], copyright 2019 American Chemical Society.

The polar groups decorated on both sides of MXene sheets render them highly hydrophilic and enable the facile intercalation of ions and polar molecules. Recently, Yang et al. [174] intercalated alkali metal ions (e.g., Na^+^) into Ti_3_C_2_T*_x_* and demonstrated its potential for humidity sensing (Figures 16(a) and 16(b)). The sensor exhibited a 60 times increase in response as the RH was increased from 33% to 95% (Figures 16(c) and 16(d)). Such good performance was attributed to the improved H_2_O adsorption in the presence of Na^+^ ions via the formation of [Na(H_2_O)_m_]^+^ clusters (Figure 16(e)). This in turn increased the amount of H_2_O molecules to transfer charges to Ti_3_C_2_T*_x_*. Besides, the intercalation-induced electronic decoupling between layers might improve the conductivity as well [175].

In fact, MXenes with intercalated ions and water molecules could be advantageous in the selective sensing of polar vapor molecules. This was demonstrated by Koh et al. [176] that laminated Ti_3_C_2_T*_x_* treated with NaOH solution exhibited a selective response toward ethanol as against CO_2_ (Figures 16(f) and 16(g)). This is because, as compared to CO_2_, ethanol molecules are more hydrophilic and easier to diffuse into the interlayer spaces in the presence of H_2_O and Na^+^. However, it is worth noting that the concentration of Na^+^ ions should not be too high; otherwise, an ordered Na^+^-H_2_O structure might form to hinder the further insertion of ethanol molecules.

#### 3.2.2. Formation of Heterostructures

**Figure 17:**
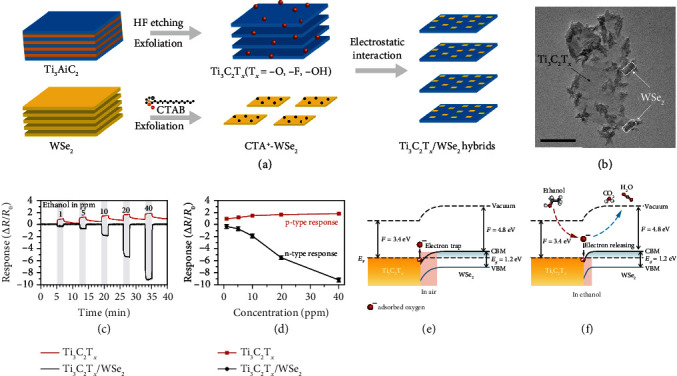
(a) Schematic illustration of preparation processes for Ti_3_C_2_T*_x_*/WSe_2_ nanohybrids. (b) Low magnification TEM image of a single Ti_3_C_2_T*_x_*/WSe_2_ nanohybrid (scale bar, 100 nm). (c) Real-time sensing response of Ti_3_C_2_T*_x_* and Ti_3_C_2_T*_x_*/WSe_2_ gas sensors upon ethanol exposure with concentrations ranging from 1 to 40 ppm. (d) Comparison of gas response as a function of ethanol gas concentrations for Ti_3_C_2_T*_x_* and Ti_3_C_2_T*_x_*/WSe_2_ sensors. Energy band diagram of Ti_3_C_2_T*_x_*/WSe_2_ (e) before and (f) after exposure to ethanol [177], copyright 2020 Nature Publishing Group.

Considering the fact that a large number of MXenes are metal-like in nature, their gas sensing performance can be enhanced by coupling them with semiconducting materials. As in a very recent work by Chen et al., an ethanol sensor based on Ti_3_C_2_T*_x_*/WSe_2_ heterostructures (Figures 17(a) and 17(b)) exhibited over 12-fold improvement of sensitivity as compared with pristine Ti_3_C_2_T*_x_* (Figures 17(c) and 17(d)) [177]. Based on their band alignment diagram (Figures 17(e) and 17(f)), an Ohmic contact is formed between Ti_3_C_2_T*_x_* and WSe_2_. Before gas adsorption, electrons in the sensing film might be trapped by preabsorbed oxygen species (O_2_^−^and O^−^) from the air, resulting in the formation of an electron depletion layer at the heterointerface. When the sensor was exposed to ethanol, the oxygen species reacted with ethanol and generated CO_2_ and H_2_O. This resulted in the release of electrons and reduction of the width of the electron depletion layer, leading to an evident drop in the film resistance.

#### 3.2.3. Effect of an External Field

**Figure 18:**
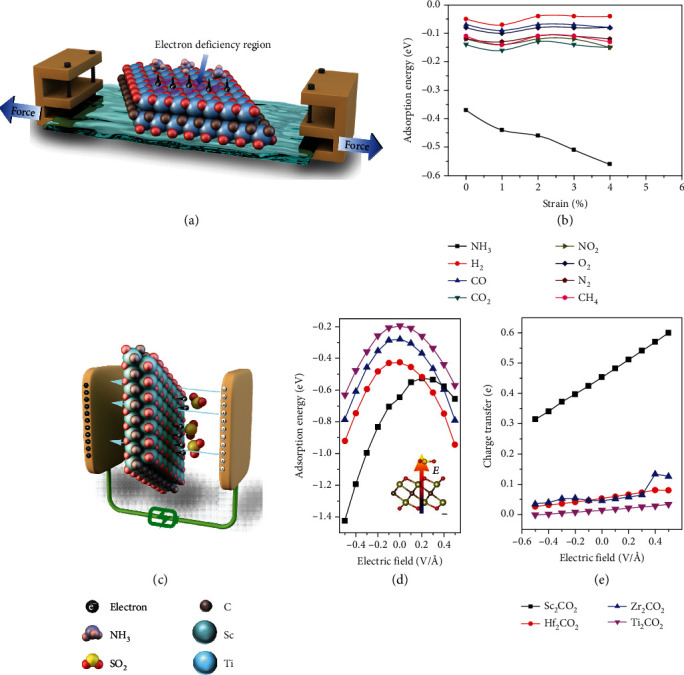
(a) Schematic illustration of applying an external stress field on a 2D gas sensing material. (b) Relationship between adsorption energies of gas molecules on the monolayer Ti_2_CO_2_ and applied biaxial strains [180], copyright 2015 American Chemical Society. (c) Schematic illustration of the effect of applying an electric field on a 2D gas sensing material. The variation of (d) adsorption energy and (e) charge transfer of SO_2_ on Sc_2_CO_2_, Hf_2_CO_2_, Zr_2_CO_2_, and Ti_2_CO_2_, as a function of the electric field strength [181], copyright 2017 American Chemical Society.

The stress field or strain imposed on a 2D material (Figure 18(a)) may change its interatomic distances and thus its crystalline and electronic structure [178]. This in turn can alter its electrical response upon gas adsorption, which is a process likely involving adsorption configuration, charge state modification, and orbital hybridization change between the gas molecules and the sensing material [179, 180]. For example, based on first-principle calculations, Yu et al. [180] found that the sensing response of Ti_2_CO_2_ toward NH_3_ could be significantly improved under tensile strain (Figure 18(b)). This is because the tensile strain could reduce the number of electrons on the Ti atoms, and such electron deficiency could strengthen the adsorption of NH_3_ on Ti_2_CO_2_ and increase the amount of charges transferred between them. Furthermore, other gases such as CO, NO_2_, and O_2_ are almost insensitive to strain, which would result in the further improved selectivity of Ti_2_CO_2_ toward NH_3_ under strain (Figure 18(b)). Similarly, Yang et al. theoretically verified that the NO adsorption behavior of Sc_2_CO_2_ could be influenced by strain [173].

In addition to the stress field, the application of an external electric field (Figure 18(c)) can also influence the interaction between the gas molecules and the sensing materials via dipole-dipole interaction [181]. Normally, the charge density distribution in a gas-solid interaction system is asymmetrical, and a built-in electric field with a net dipole moment will be generated [182]. The larger the dipole moment, the larger the amount of the exchanged electrons between the gas molecules and the sensing material [183]. An external electric field could thus offset or enhance the built-in electric field and therefore is able to control the direction and quantity of the charge transfer [182, 184, 185]. In a recent theoretical investigation based on first-principle calculations, Ma et al. [181] reported that the adsorption energy and charge transfer of SO_2_ on Sc_2_CO_2_ could be modulated by applying an electric field (Figures 18(d) and 18(e)). It is interesting that the amount of charge transferred from Sc_2_CO_2_ to SO_2_ increases linearly as the electric field is increased from negative to positive, a trend that is less obvious in other systems involving Hf_2_CO_2_, Zr_2_CO_2_, and Ti_2_CO_2_ (Figure 18(d)). This is attributed to the stronger interaction between SO_2_ and Sc_2_CO_2_ due to the intrinsic net dipole moment present in the monolayer Sc_2_CO_2_. It is also important to note that the external field should not be too high to ensure easy desorption of gas molecules.

## 4. Black Phosphorus

Layer-structured black phosphorus (BP) was previously prepared from white phosphorus through high-temperature and high-pressure treatments [187]. Alike graphene, monolayers of BP stack together via the van der Waals interaction to form layered BP crystals [188]. Isolated single-layer BP, also termed phosphorene, was firstly prepared via the scotch tape-based microcleavage method [189]. The hole mobility of bulk BP is about 1000 cm^2^ V^−1^ s^−1^, and that for phosphorene can reach up to 10,000 cm^2^ V^−1^ s^−1^ [190–192]. Similar to the other 2D atomic crystals, BP possesses the thickness-dependent bandgap energy, which varies from ~1.5 eV for the monolayer to ~0.3 eV for the bulk BP [29, 188]. This correlates to light adsorption covering wavelengths ranging from the near-infrared to middle-infrared bands which is in between that of graphene and that of MCs [193, 194]. These advantages make BP extremely attractive for electronics and optoelectronics, and therefore, they have been extensively explored in applications such as memories [195, 196], photodetectors [197, 198], and sensors [199, 200].

For gas sensing, in particular, BP has demonstrated several attractive features. For example, BP possesses a high theoretical surface-to-volume ratio due to its puckered double-layer structure, which can offer abundant adsorption sites for target analytes [201]. Besides, BP has been predicted to possess high adsorption energies toward many small gas molecules such as NO_2_, NO, NH_3_, CO, and SO_2_ [202–204]. In the following contents, we introduce BP-based gas sensors, emphasize their working principles involving charge transfer, surface reaction, and mass change, and describe strategies used to enhance their gas sensing performance (Table 3).

### 4.1. Sensing Mechanisms

#### 4.1.1. Sensing via Charge Transfer

**Figure 19:**
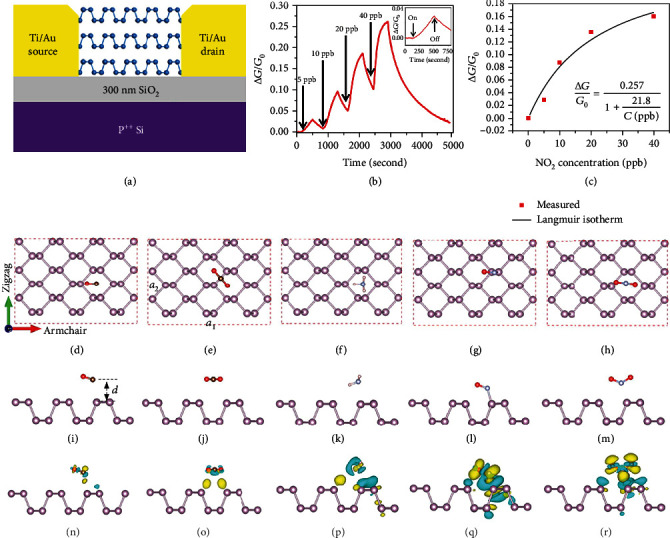
(a) Scheme of a multilayer BP FET. (b) Relative conductance change (Δ*G*/*G*_0_) vs. time in seconds for a multilayer BP sensor showing sensitivity to NO_2_ concentrations (5-40 ppb). Inset shows a zoomed-in image of a 5 ppb NO_2_ exposure response with identification of points in time where the NO_2_ gas is switched on and off. (c) Δ*G*/*G*_0_ plotted vs. NO_2_ concentration applied to the BP FET showing an agreement between the measured values (red squares) and the fitted Langmuir isotherm. The equation in the bottom right is the fitted Langmuir isotherm [199], copyright 2015 American Chemical Society. Top view (d–h) and side view (i–m) of the fully relaxed structural models of phosphorene with CO, CO_2_, NH_3_, NO, and NO_2_ adsorption, respectively. The red dashed rectangle is the supercell. The brown balls represent P atoms, while the black, red, cyan, and light white balls indicate C, O, N, and H atoms, respectively. The adsorption configurations and charge transfer for each case are plotted in (n–r) with CO, CO_2_, NH_3_, NO, and NO_2_ adsorption, respectively. The isosurface value for all of the cases is 10^−3^ e/Å^3^. The yellow isosurface indicates an electron gain, while the blue one represents an electron loss [202], copyright 2014 American Chemical Society.

Alike MCs and MXenes, charge transfer is the dominant gas sensing mechanism for BP. In an early demonstration, a multilayer p-type BP-based FET NO_2_ sensor was fabricated by Abbas et al., and a minimum detectable concentration down to 5 ppb was achieved (Figures 19(a) and 19(b)) [199]. The change of conductance as a function of NO_2_ concentration could be fitted well with the Langmuir isotherm, suggesting that the NO_2_ adsorption-induced charge transfer is the dominant cause of the conductance change (Figure 19(c)). It was later pointed out by Cho and coworkers that BP exhibited a higher adsorption energy for NO_2_ as compared to graphene and MoS_2_ based on DFT calculations [205], and they experimentally demonstrated the superior sensing performance of BP in terms of sensitivity, response time, and selectivity [205].

A systematic DFT calculation study by Kou et al. reveals the different adsorption behaviors of several gas molecules including CO, CO_2_, NH_3_, NO, and NO_2_ on BP (Figures 19(d)–19(r)) [202]. The carbon atoms of CO molecules and nitrogen atoms of NH_3_ molecules are likely located at the center of the puckered honeycomb of BP, whereas CO_2_ molecules are adsorbed at the bridges of the P-P bonds. Among these gases, only NO interacts with BP via the formation of P-N bonds, whereas other gases stay above the basal layer at a distance without bond formation. Their results also indicate larger adsorption energies and enhanced charge transfer of N-based gases (e.g., NO*_x_* and NH_3_) on BP as compared to CO and CO_2_.

**Figure 20:**
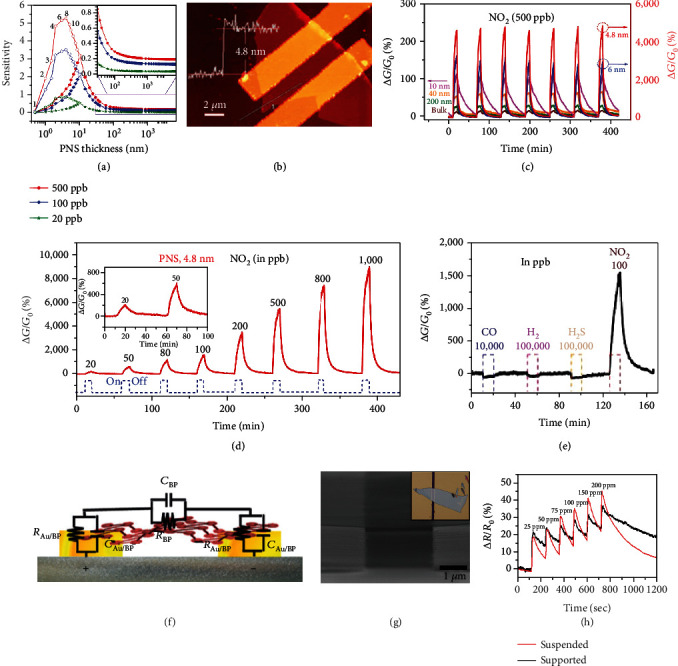
(a) Simulation result of the sensitivity of the BP nanosheet sensor as a function of the nanosheet thickness at different NO_2_ concentrations. The hollow symbols indicate cases when the effect of mobility degradation is not considered. The inset is the zoomed view of the sensitivity for the BP nanosheet thickness from 30 nm to the bulk. (b) Atomic force microscopy (AFM) images of the BP nanosheet sensor. (c) Thickness-dependent multicycle responses of the BP nanosheet sensor to 500 ppb NO_2_. (d) Dynamic response curves of relative conductance change versus time for NO_2_ concentrations ranging from 20 to 1000 ppb (balanced in dry air) for the BP nanosheet (4.8 nm). A drain-source voltage of 0.6 V was applied to the device. The dashed line demonstrates the “on/off” of NO_2_ gas. The sensitivity here is defined as the differential response between Δ*G*/*G*_0_ = 0 in the air environment at the first cycle and the Δ*G*/*G*_0_ at the end of gas “off” for each concentration. (e) Dynamic sensing response curve of the 4.8 nm PNS to various gases, including 10,000 ppb CO, 100,000 ppb H_2_, 10,000 ppb H_2_S, and 100 ppb NO_2_. The sensor shows a much higher response to NO_2_ compared with other gases [29], copyright 2015 Nature Publishing Group. (f) Equivalent circuit for the BP-based vapor sensor [208], copyright 2015 John Wiley & Sons, Inc. (g) Scanning electron microscopy (SEM) images of the suspended BP flakes. (h) Responses of the supported and suspended BP sensors toward NO_2_ [213], copyright 2017 Elsevier Ltd.

From the previous knowledge gained from graphene and MC-based gas sensors, and considering the fact that BP possesses a direct bandgap that increases when reducing the layer number [189, 206], it can be expected that BP also exhibits the thickness-dependent sensing behavior. Cui and coworkers suggested that a thinner BP nanosheet with a larger bandgap and less intrinsic carriers might transfer less charges to a target gas, e.g., NO_2_. However, a thicker BP with a smaller bandgap would undergo less change of conductivity due to its high carrier concentration [29]. Therefore, an optimum thickness exists to maximize the gas sensing performance. They predicted that nanosheets with 4.3-10 nm thickness could deliver the best sensitivity (Figure 20(a)). This agrees with their experimental result: the sensor based on ~4.8 nm BP nanosheets exhibited higher responses toward sub-ppm levels of NO_2_ than those based on bulk BP and BP with thicknesses ranging from 6 to 200 nm (Figures 20(b)–20(d)), along with an extremely good selectivity (Figure 20(e)).

The gas adsorption-induced charge transfer can be reflected, in addition to the resistance change mentioned above, also as a change of impedance [207]. As an example, layered BP nanosheets were used to sense methanol vapor via electrochemical impedance spectroscopy (EIS) measurements by Mayorga-Martinez and coworkers [208]. The sensor showed high selectivity and sensitivity toward 28 ppm methanol under an alternative current (AC) frequency of 1 kHz. The equivalent circuit is shown in Figure 20(f), which consists of the resistance and capacitance components. The resistance change was attributed to the charge transfer from the target gas to BP [209], whereas the capacitance change arose from the change of dielectric constants at the Au electrode/BP interface, which is related to the number of absorbed gas molecules based on polarization [210]. For example, methanol and ethanol have different dielectric constants of 32.7 and 24.6 and different resonance frequencies of ~10^3^ Hz and over 10^5^ Hz, respectively. Such difference enables the selective detection of methanol realized at 10^3^ Hz [208].

Similar to many semiconductor electronic devices, the supporting substrate such as SiO_2_/Si for a BP-based sensing film can introduce dopants, scattering centers, or traps that can modify BP's electrical transport properties and influence its sensing performance [211, 212]. To alleviate the effect of the substrate, Lee and colleagues fabricated a gas sensor by placing a BP nanoflake over a wet-etched SiO_2_ trench in a SiO_2_/Si substrate (Figure 20(g)) [213]. Due to the elimination of charge scattering from the substrate and the increased gas adsorption sites on both sides of a suspended BP nanoflake, the sensor showed a 23% higher response and a two times faster rate of desorption toward 200 ppm NO_2_ as compared with BP supported on the substrate (Figure 20(h)).

#### 4.1.2. Surface Reaction

**Figure 21:**
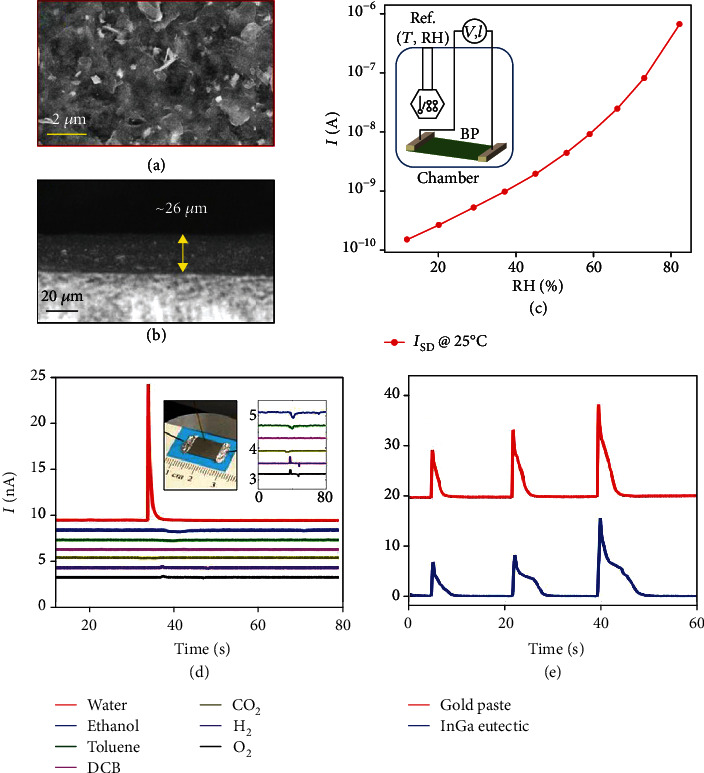
(a) Top and (b) cross-section images of the film of stacked BP nanoflakes. (c) The current of a typical BP sensor vs. RH at 25°C. (d) Response of the stacked BP nanoflakes to different analytes. (e) Comparative pulse injection sensing response of BP films with different gold paste electrodes and InGa electrodes [218], copyright 2015 American Chemical Society.

The surface hydrophilicity of BP and its susceptibility to surface reactions have enabled its application in humidity sensing [214]. The charge transfer from water to BP has been suggested by several groups as the main working principle for BP-based humidity sensors [32, 215, 216]. First-principle calculations by Cai and coworkers also showed that each H_2_O molecule could donate about 0.035 e to BP [217]. However, the investigation carried out by Yasaei et al. suggested otherwise. They fabricated a BP sensor capable of achieving ∼4 orders of the current increase as the RH was varied from 10% to 85% (Figures 21(a)–21(c)) [218]. Interestingly, this humidity sensor showed very high selectivity, evidenced by the orders of lower response toward common polar gases, such as ethanol, toluene, and dichlorobenzene (DCB). This also suggests that the charge transfer should not be the main reason behind the high sensing response toward water vapor (Figure 21(d)). In addition, the effect from the BP/electrode contact was also excluded, because the sensor with InGa eutectic and Au electrodes showed a similar sensing response (Figure 21(e)). They thus proposed that when the BP flakes were exposed to humid air, they could react with water to form phosphorus oxoacids. The autoionization of water and ionic dissolution of the acid could generate a large number of mobile ions and thereby decrease the resistance of the BP channel.

#### 4.1.3. Sensing via Mass Change

**Figure 22:**
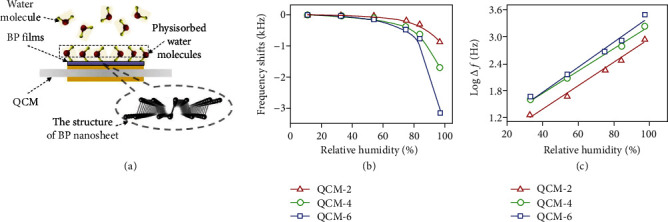
(a) Schematic diagram of the water adsorption model of the BP-based QCM humidity sensors. (b) The frequency response of the BP-based QCM humidity sensors as a function of humidity. (c) The logarithmic fitting curves of log (Δ*f*) versus humidity for all the sensors [31], copyright 2017 Elsevier Ltd.

As mentioned in Introduction, BP can be fabricated into QCM-based humidity sensors because of the high affinity of BP for water molecules. For example, Yao et al. [31] fabricated a QCM-based humidity sensor by depositing BP nanosheets onto the electrode surface of a QCM (Figure 22(a)). The change in the resonance frequency (Δ*f*) of the quartz correlates to the amount of water molecules adsorbed on BP. A large response, i.e., 863 Hz, 1698 Hz, and 3145 Hz of the frequency shift for the three sensors fabricated with an increasing amount of BP (QCM-2, QCM-4, and QCM-6 correspond to 2, 4, and 6 *μ*L of BP nanosheets deposited on the electrode), respectively, was achieved as the humidity was increased from 11.3% to 97.3% (Figures 22(b) and 22(c)). Such a high response was also accompanied by a fast response and recovery time, good repeatability, and long-term stability. The selectivity of this type of gas sensor depends on the specific adsorption of BP toward water molecules.

### 4.2. Approaches to Enhance Gas Sensing Performance of BP

**Figure 23:**
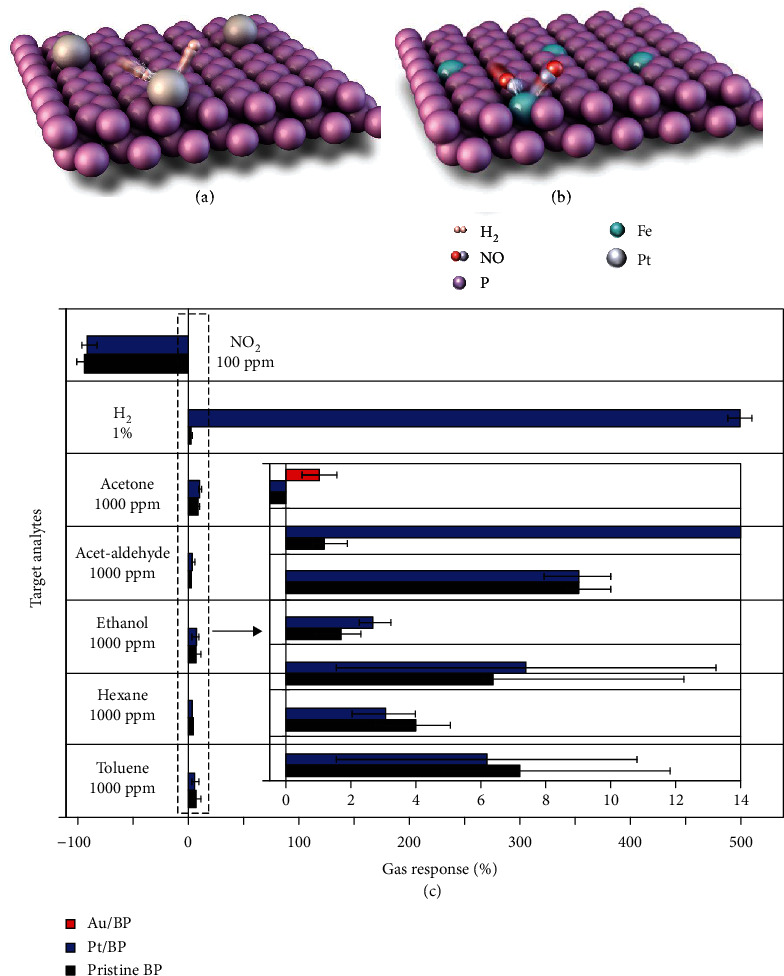
Schematic illustration of (a) decoration of Pt NPs and (b) doping with Fe atoms to enhance sensing performance. (c) Summary of gas response of the pristine BP, Au/BP, and Pt/BP for various target chemical analytes [224], copyright 2017 American Chemical Society.

Very recently, to enhance the sensing performance of BP, strategies such as the incorporation of metal nanoparticles (NPs) and adatom doping have been explored (Figures 23(a) and 23(b)) [219, 220]. Generally speaking, the effects of NP decoration on a sensor's performance can be twofold, that is, the electronic sensitization effect and the chemical sensitization effect. The former is related to the modulated carrier concentration and mobility, whereas the latter is related to the improved binding affinity for the target gas [221]. Lee et al. demonstrated that pristine BP was insensitive to H_2_, while Pt NP-functionalized BP nanosheets exhibited high sensitivity to H_2_ [219]. It is a well-accepted fact that H_2_ adsorbed on Pt can form PtH*_x_* which has a lower work function than Pt [222]. This can result in the spontaneous electron transfer from PtH*_x_* to the p-type BP to reduce its conductance [223]. A similar beneficial effect from Pt NP functionalization was reported by Cho et al. [224], demonstrating ultrahigh selectivity in sensing H_2_ (Figure 23(c)). They also found that the decoration of Au NPs on BP nanosheets could convert BP from the p-type semiconductor to the n-type semiconductor by donating extra electrons. This led to an increased resistance upon exposure to oxidizing gases, such as NO_2_.

Besides modulating the carrier concentration [225, 226], doping BP with adatoms such as Fe, Ni, and Al is able to enhance its binding affinity for certain gases based on theoretical predictions [220, 226, 227]. For example, the binding energies for NO and CO on BP are -0.32 eV and 0.12 eV, respectively, and increase to -2.58 eV on Fe-doped BP [220] and to -1.16 eV on Ni-doped BP [227], respectively.

## 5. Summary and Outlooks

2D materials are promising building blocks in the future development of various sensing platforms. In this review, by looking into the recent experimental and theoretical investigations of the gas sensing behaviors of 2D atomic crystals such as MCs, BP, and MXenes, we have discussed their gas sensing mechanisms and the related influential factors, as well as adoptable approaches that can enhance their sensing performance (Table 4).

Direct gas adsorption-induced charge transfer has been found to be the dominating gas sensing mechanism in many 2D material-based electrical gas sensors such as chemiresistive and FET sensors. Alike conventional semiconducting metal oxide gas sensors, reactions between target gas molecules and preabsorbed oxygen species and water molecules have also been found to occur on the surfaces of MCs or BPs under ambient conditions or at slightly raised temperatures, which induce charge transfer and conductivity change. When the relative humidity becomes high, especially when water molecules are the target for sensing, continuous H^+^/H_3_O^+^ pathways on the 2D material surfaces may form which contribute further to the conductivity change via the proton conduction mechanism. MXenes show special gas sensing behaviors in contrast to most semiconducting MCs and BPs, because many of them are intrinsically metal-like in nature with narrow bandgaps. Therefore, in MXene-based gas sensors, the gas adsorption-induced charge scattering presents another important sensing mechanism in addition to the charge transfer mechanism; which mechanism is dominant depends on the composition, surface chemistry, and dopant type/concentration of the MXene, as well as the working temperature.

Common factors such as the layer number, surface functionalities, electron channel contact, gate bias, and working temperature have been found to influence the gas sensing behaviors of 2D materials. Again, MXenes are special in a way that their terminal groups, which can be controlled by the synthesis and treatment methods, can exert profound effects on their band structures and therefore sensing pathways.

Further enhancement of the gas sensing performance of the various 2D material-based devices, in terms of sensitivity, selectivity, and response/recovery time, can be achieved via the creation of 3D porous networks, adatom doping/ion intercalation, formation of composites/heterostructures, and application of light, stress field, or electrostatic field. For example, the creation of 3D porous networks can enlarge gas-solid interaction surfaces and improve gas adsorption/desorption kinetics; the formation of heterostructures can modulate charge transport barriers; and light activation can increase the charge carrier concentration and even modify the molecular orbital structure of the target gas.

Understanding the sensing mechanisms involving surface processes is of paramount importance in the further development of advanced sensing materials and device structures. This requires advanced surface characterization techniques, especially those capable of in situ monitoring. For example, synchrotron-based in situ time-resolved X-ray diffraction and X-ray absorption spectroscopy can help characterize the chemical states of the initial, intermediate, and final structure of a sensing material during the sensing process, which is favorable for speculating the sensing process and analysis of the sensing mechanism.

As the map of 2D atomic crystals continues to expand over the years, emerging 2D materials such as borophene, arsenene, antimonene, and BiOSe [1, 228–231] are also promising candidates for gas sensing. For example, borophene, a single layer of boron atoms, and related derivatives have been prepared recently [232–236]. A number of theoretical investigations have shown their potential applications in the chemiresistive type of gas sensors for VOCs such as ethanol [237], formaldehyde [238], acetaldehyde [239], and propanal [239], as well as uncommon toxic gases such as HCN [240], COCl_2_, and CO [241]. These novel 2D material-based gas sensing systems require further experimental exploitations.

It is however the common obstacle facing most gas sensing nanomaterials that they show limited selectivity. Although preferred gas adsorption, surface reaction, intercalation, and carrier doping have rendered these 2D materials with a certain level of selectivity, there is still big room for improvement when compared with spectroscopy-based sensing systems. Besides, for room temperature sensors, the interference from the variation of ambient temperature and relative humidity is unneglectable and can largely affect the sensor selectivity. The fabrication of sensor arrays combined with data processing has been an effective strategy to improve sensing selectivity and has been used in many commercial electrical sensors. Very recently, single-atom catalysts (SACs) have been prepared on metal oxide via surface coordination to improve the selectivity and sensitivity of room temperature gas sensors, which can also be adopted for 2D material-based gas sensors. For example, atomic Pd can provide a higher adsorption efficiency for oxygen species which in turn enhances the sensitivity and selectivity toward CO [242]. In addition, as compared with a single-output sensor, which is the case for most reported 2D material-based electrical sensors, a gas sensor with multivariant signal output is capable of achieving better selectivity and reliability [243]. For example, a suitable 2D crystal can be integrated into a device that can output electrical resistance change as well as resonance frequency change as in a QCM setup. This actually demands strongly on the future exploration of smart gas sensing materials.

Durability is a key issue facing practical gas sensors. This becomes particularly challenging when surface reactions are involved in the sensing process in the presence of humid air at elevated working temperatures. Surface incorporation with metal NPs to induce catalytic reaction instead of direct reaction between the gas molecules and the 2D material might help improve the sensor stability. In addition, the use of a humidity filter or functionalization of the 2D material with molecules with hydrophobic terminal groups can reduce the effect of humidity; however, this may also reduce sensitivity and increase response time. Besides, the incorporation of 2D materials in the optic fiber and photonic crystal gas sensors may offer enhanced durability as compared to chemiresistive gas sensors.

One of the advantages of 2D materials lies in their intrinsic flexibility which enables their integration into wearable and portable gas sensors for applications such as real-time environmental monitoring [244] and analysis of breath content for health monitoring [27]. Although much progress has been made in gas sensing with 2D materials, wearable and portable gas sensors have shown limited development. This is largely due to the reduced sensitivity and durability under bending or stretched conditions because both the mechanical strain and gas adsorption can change the sensing response. Such crosstalk problem might be tackled by proper structure design and use of sensor arrays with data processing.

Currently, most commercially available gas sensors are based on metal oxides which function at elevated temperatures. Gas sensors based on 2D materials are able to deliver enhanced sensitivity at room temperature at reduced power consumption. However, their sensing responses at low gas concentrations are still not high enough such that they are prone to environmental perturbations. In other words, factors such as humidity, temperature, and strain may cause unneglectable changes to the sensing response and affect their reliability and durability. Therefore, there is an urgent need to explore novel 2D gas sensing materials with high sensitivity, selectivity, and stability and develop new strategies to further improve the sensitivity of room temperature 2D material gas sensors.

## Figures and Tables

**Table 1:** Selected examples of gas sensors based on MCs.

Target gas	Materials	Mechanism	Detection limit/minimum detected concentration	Response/sensitivity	Temperature	Type	Dopant type	Ref.
NO_2_	4 nm MoS_2_	Charge transfer	1.2 ppm	6.1% @ 1.2 ppm	RT	FET	n-type	[83]
	5-layer MoS_2_	Charge transfer	/	1372% @ 1000 ppm	RT	FET	n-type	[55]
	MoS_2_ nanowire network	Charge transfer	4.6 ppb/1 ppm	18.1% @ 5 ppm	60°C	Chemiresistor	n-type	[62]
	Vertically aligned MoS_2_ layers	Charge transfer	~2.3 ppb/1 ppm	16.2% @ 1 ppm	RT	Chemiresistor	n-type	[60]
	MoS_2_	Charge transfer	/	98% @ 10 ppm	RT	Chemiresistor	p-type	[80]
	MoS_2_	Charge transfer	0.1 ppb/25 ppb	4.9% @ 1 ppb	RT	Chemiresistor	n-type	[64]
	Monolayer MoS_2_	Dipole scattering	0.15	8.6% @ 1 ppb	RT	FET	n-type	[126]

NH_3_	5-layer MoS_2_	Charge transfer	/	86% @ 1000 ppm	RT	FET	n-type	[55]
	Single-layer WS_2_	Charge transfer	50 ppm	3.14% @ 500 ppm	RT	Chemiresistor	n-type	[61]
	WS_2_ nanosheets	Charge transfer	5 ppm	3.4% @ 10 ppm	RT	Chemiresistor	p-type	[117]

Ethanol	MoSe_2_ nanosheets	Charge transfer	10 ppm	~18% @ 20 ppm	90°C	Chemiresistor	p-type	[57]

Ketone	MoTe_2_	Charge transfer	0.2 ppm	~58% @ 100 ppm	RT	FET	p-type	[59]

Propionaldehyde	MUA-conjugated MoS_2_	Charge transfer	A few ppm	/	RT	Chemiresistor	n-type	[112]
Acetone		Charge transfer	A few ppm	/				
Toluene		Charge transfer	10 ppm	/				
Hexane		Charge transfer	10 ppm	/				
Ethanol		Charge transfer	100 ppm	/				

H_2_S	WS_2_ nanowire-nanoflake hybrid	Surface reaction	20 ppb	4.3% @ 1 ppm	200°C	Chemiresistor	p-type	[68]

NO_2_	Hierarchical hollow MoS_2_ microspheres	Surface reaction	0.5 ppm	40.3% @ 100 ppm	150°C	Chemiresistor	p-type	[116]

NH_3_	WS_2_ nanoflakes	Surface reaction	1 ppm	~900% @ 10 ppm	RT	Chemiresistor	p-type	[67]

Humidity	MoS_2_/graphene oxide nanocomposite	Proton conduction	/	~200% @ 45% RH	RT	Chemiresistor	n-type	[65]

NO_2_	Ultrathin WS_2_ nanosheets	/	0.1 ppm	9.3% @ 0.1 ppm	25°C	Chemiresistor	p-type	[70]
	NbSe_2_/WSe_2_	/	0.12 ppm	/	/	Chemiresistor	/	[136]
	BP/MoSe_2_	/	10 ppb	~10.5% @ 25 ppb	RT	FET	/	[74]
	Graphene/MoS_2_ composite	/	/	61% @ 500 ppm	RT	Fiber optic	Refractive index	[105]

Humidity	WS_2_/WSe_2_	/	/	57 times @ 80% RH	RT	Chemiresistor	/	[141]

Acetone	Sn_0.5_W_0.5_S_2_/SnS_2_	/	0.1 ppm	0.60% @ 0.4 ppm	RT	Chemiresistor	/	[137]

Methanol	MoS_2_	/	2.7 ppm	0.37 pm @ ppm	RT	Photonic crystal	/	[107]

**Table 2:** Selected examples of gas sensors based on MXenes.

Materials	Target gas	Detection limit/minimum detected concentration	Response/sensitivity	Temperature	Type	Mechanism	Ref.
Ti_3_C_2_T*_x_*	Acetone	0.011 ppb	0.97% @ 100 ppm	RT	Chemiresistor	Scattering	[152]
	Ethanol	Sub-ppb	1.7% @ 100 ppm				
	NH_3_	0.13 ppb	0.8% @ 100 ppm				
	Propanal	/	0.88% @ 100 ppm				

Ti_3_C_2_T*_x_*	Acetone	9.27 ppm	0.075% @ 100 ppm	RT	Chemiresistor	Charge transfer	[27]
	Ethanol	/	0.115% @ 100 ppm				
	Methanol	/	0.143% @ 100 ppm				
	NH_3_	/	0.21% @ 100 ppm				

Ti_3_C_2_T*_x_*	Ethanol	/	9.995% @ 1000 ppm	RT	Chemiresistor	Charge transfer	[176]

Ti_3_C_2_T*_x_*	Humidity	/	60 times response change @ 11−95% RH	RT	Chemiresistor	Charge transfer	[174]
	NH_3_	/	28.87% @ 100 ppm				

3D Ti_3_C_2_T*_x_*	Acetone	50 ppb	0.10~0.17 @ ppm	RT	Chemiresistor	Charge transfer	[186]
	Ethanol						
	Methanol						
	NH_3_						

Ti_3_C_2_T*_x_*/WSe_2_ hybrid	Ethanol	1 ppm	0.24% @ ppm	RT	Chemiresistor	Charge transfer	[177]

**Table 3:** The comparison of the sensors based on BP.

Materials	Target gas	Response	Detection limit/minimum detected concentration	Temperature	Type	Ref.
BP	NO_2_	1600% @ 20 ppb	20 ppb	RT	FET	[29]
BP	NO_2_	2.9% @ 5 ppb	5 ppb	/	FET	[199]
BP	NO_2_	80% @ 1 ppm	0.1 ppm	RT	Chemiresistor	[205]
BP	NH_3_	13% @ 10 ppm	80 ppb	RT	Chemiresistor	[188]
BP	Humidity	~4 orders @ 10%-85% RH	10% RH	RT	Chemiresistor	[218]
BP	Humidity	~521% @ 97% RH	/	25°C	FET	[215]
BP	Humidity	99.17% @ 97.3% RH	11.3% RH	RT	Chemiresistor	[216]
BP	Humidity	3145 Hz @ 97.3% RH	11.3% RH	RT	QCM	[31]
Pt/BP	H_2_	50% @ 4%	500 ppm	RT	FET	[219]
Pt/BP	H_2_	500% @ 1%	10 ppm	RT	Chemiresistor	[224]

**Table 4:** An overview of 2D atomic crystals with high sensing performance in this review.

Target gas	Materials	Detection limit	Response/sensitivity	Temperature	Type	Ref.
NO_2_	4 nm MoS_2_	1.2 ppm	6.1% @ 1.2 ppm	RT	FET	[83]
	BP/MoSe_2_	10 ppb	~10.5% @ 25 ppb	RT	FET	[74]
	BP	5 ppb	2.9% @ 5 ppb	/	FET	[199]
	Monolayer MoS_2_	0.15 ppb	8.6% @ 1 ppb	RT	FET	[126]

NH_3_	WS_2_ nanoflakes	1 ppm	~900% @ 10 ppm	RT	Chemiresistor	[67]
	3D Ti_3_C_2_T*_x_*	50 ppb	0.10~0.17 @ ppm	RT	Chemiresistor	[186]
	BP	80 ppb	13% @ 10 ppm	RT	Chemiresistor	[205]

H_2_	Pt/BP	500 ppm	50% @ 4%	RT	FET	[219]
	Pt/BP	10 ppm	500% @ 1%	RT	Chemiresistor	[224]

Humidity	MoS_2_/graphene oxide nanocomposite	/	~200% @ 4 5% RH	RT	Chemiresistor	[65]
	BP	11.3% RH	3145 Hz @ 97.3% RH	RT	QCM	[31]
	Ti_3_C_2_T*_x_*	/	60 times response change @ 11-95% RH	RT	Chemiresistor	[174]

Ethanol	MoSe_2_ nanosheets	10 ppm	~18% @ 20 ppm	90°C	Chemiresistor	[57]
	3D Ti_3_C_2_T*_x_*	50 ppb	0.10~0.17 @ ppm	RT	Chemiresistor	[186]

Acetone	Sn_0.5_W_0.5_S_2_/SnS_2_	0.1 ppm	0.60% @ 0.4 ppm	RT	Chemiresistor	[245]
	Ti_3_C_2_T*_x_*	0.011 ppb	0.97% @ 100 ppm	RT	Chemiresistor	[152]

Methanol	3D Ti_3_C_2_T*_x_*	50 ppb	0.10~0.17 @ ppm	RT	Chemiresistor	[186]
	Ti_3_C_2_T*_x_*	/	0.143% @ 100 ppm	RT	Chemiresistor	[27]
	MoS_2_	2.7 ppm	0.37pm @ ppm	RT	Photonic crystal	[107]

## References

[1] Tang X., Du A., Kou L. (2018). Gas sensing and capturing based on two-dimensional layered materials: overview from theoretical perspective. *Wiley Interdisciplinary Reviews: Computational Molecular Science*.

[2] Anichini C., Czepa W., Pakulski D., Aliprandi A., Ciesielski A., Samorì P. (2018). Chemical sensing with 2D materials. *Chemical Society Reviews*.

[3] Liu X., Cheng S., Liu H., Hu S., Zhang D., Ning H. (2012). A survey on gas sensing technology. *Sensors*.

[4] Dorman F. L., Whiting J. J., Cochran J. W., Gardea-Torresdey J. (2010). Gas chromatography. *Analytical Chemistry*.

[5] Hamilton L. H. (1962). Gas chromatography for respiratory and blood gas analysis. *Annals New York Academy of Sciences*.

[6] Venkatasubramanian A., Sauer V. T., Roy S. K., Xia M., Wishart D. S., Hiebert W. K. (2016). Nano-optomechanical systems for gas chromatography. *Nano Letters*.

[7] Pedersen-Bjergaard S., Semb S. I., Vedde J., Brevik E. M., Greibrokk T. (1996). Environmental screening by capillary gas chromatography combined with mass spectrometry and atomic emission spectroscopy. *Chemosphere*.

[8] Ragunathan N., Krock K. A., Klawun C., Sasaki T. A., Wilkins C. L. (1999). Gas chromatography with spectroscopic detectors. *Journal of Chromatography A*.

[9] Vinaixa M., Schymanski E. L., Neumann S., Navarro M., Salek R. M., Yanes O. (2016). Mass spectral databases for LC/MS- and GC/MS-based metabolomics: state of the field and future prospects. *Trac-Trends in Analytical Chemistry*.

[10] Dominguez R., Purrinos L., Perez-Santaescolastica C. (2019). Characterization of volatile compounds of dry-cured meat products using HS-SPME-GC/MS technique. *Food Analytical Methods*.

[11] Knebl A., Yan D., Popp J., Frosch T. (2018). Fiber enhanced Raman gas spectroscopy. *Trac-Trends in Analytical Chemistry*.

[12] Hanf S., Bogozi T., Keiner R., Frosch T., Popp J. (2015). Fast and highly sensitive fiber-enhanced Raman spectroscopic monitoring of molecular H_2_ and CH_4_ for point-of-care diagnosis of malabsorption disorders in exhaled human breath. *Analytical Chemistry*.

[13] Wang P., Chen W., Wan F., Wang J., Hu J. (2019). A review of cavity-enhanced Raman spectroscopy as a gas sensing method. *Applied Spectroscopy Reviews*.

[14] Liu H., Weng L., Yang C. (2017). A review on nanomaterial-based electrochemical sensors for H_2_O_2_, H_2_S and NO inside cells or released by cells. *Microchimica Acta*.

[15] Chiu S. W., Tang K. T. (2013). Towards a chemiresistive sensor-integrated electronic nose: a review. *Sensors*.

[16] Zhao X., Cai B., Tang Q., Tong Y., Liu Y. (2014). One-dimensional nanostructure field-effect sensors for gas detection. *Sensors*.

[17] Fattah A., Khatami S. (2014). Selective H_2_S gas sensing with a graphene/n-Si Schottky diode. *IEEE Sensors Journal*.

[18] Korotcenkov G., Cho B. K. (2017). Metal oxide composites in conductometric gas sensors: achievements and challenges. *Sensors and Actuators B: Chemical*.

[19] Suehiro J., Zhou G., Imakiire H., Ding W., Hara M. (2005). Controlled fabrication of carbon nanotube NO_2_ gas sensor using dielectrophoretic impedance measurement. *Sensors and Actuators B: Chemical*.

[20] Yu D., Li J., Wang T. (2020). Black phosphorus all-fiber sensor for highly responsive humidity detection. *Physica Status Solidi-Rapid Research Letters*.

[21] Gayraud N., Kornaszewski Ł. W., Stone J. M. (2008). Mid-infrared gas sensing using a photonic bandgap fiber. *Applied Optics*.

[22] Wang C., Yin L., Zhang L., Xiang D., Gao R. (2010). Metal oxide gas sensors: sensitivity and influencing factors. *Sensors*.

[23] Wong Y. C., Ang B. C., Haseeb A. S. M. A., Baharuddin A. A., Wong Y. H. (2019). Review-conducting polymers as chemiresistive gas sensing materials: a review. *Journal of the Electrochemical Society*.

[24] Han T., Nag A., Mukhopadhyay S. C., Xu Y. Z. (2019). Carbon nanotubes and its gas-sensing applications: a review. *Sensors and Actuators A: Physical*.

[25] Varghese S. S., Varghese S. H., Swaminathan S., Singh K. K., Mittal V. (2015). Two-dimensional materials for sensing: graphene and beyond. *Electronics*.

[26] Liu X., Ma T., Pinna N., Zhang J. (2017). Two-dimensional nanostructured materials for gas sensing. *Advanced Functional Materials*.

[27] Lee E., VahidMohammadi A., Prorok B. C., Yoon Y. S., Beidaghi M., Kim D.-J. (2017). Room temperature gas sensing of two-dimensional titanium carbide (MXene). *ACS Applied Materials & Interfaces*.

[28] Donarelli M., Ottaviano L. (2018). 2D materials for gas sensing applications: a review on graphene oxide, MoS_2_, WS_2_ and phosphorene. *Sensors*.

[29] Cui S., Pu H., Wells S. A. (2015). Ultrahigh sensitivity and layer-dependent sensing performance of phosphorene-based gas sensors. *Nature Communications*.

[30] Polo J., Llobet E., Vilanova X., Brezmes J., Correig X. (1999). SPICE model for quartz crystal microbalance gas sensors. *Electronics Letters*.

[31] Yao Y., Zhang H., Sun J. (2017). Novel QCM humidity sensors using stacked black phosphorus nanosheets as sensing film. *Sensors and Actuators B: Chemical*.

[32] Korotcenkov G. (2019). Black phosphorus-new nanostructured material for humidity sensors: achievements and limitations. *Sensors*.

[33] Chen W., Deng F. F., Xu M., Wang J., Wei Z., Wang Y. (2018). GO/Cu_2_O nanocomposite based QCM gas sensor for trimethylamine detection under low concentrations. *Sensors and Actuators B: Chemical*.

[34] Zhao Y., Zhang Y. N., Wang Q. (2012). High sensitivity gas sensing method based on slow light in photonic crystal waveguide. *Sensors and Actuators B: Chemical*.

[35] Wang T., Huang D., Yang Z. (2016). A review on graphene-based gas/vapor sensors with unique properties and potential applications. *Nano-Micro Letters*.

[36] He Q., Wu S., Yin Z., Zhang H. (2012). Graphene-based electronic sensors. *Chemical Science*.

[37] Gupta Chatterjee S., Chatterjee S., Ray A. K., Chakraborty A. K. (2015). Graphene-metal oxide nanohybrids for toxic gas sensor: A review. *Sensors and Actuators B: Chemical*.

[38] Yang S., Jiang C., Wei S. (2017). Gas sensing in 2D materials. *Applied Physics Reviews*.

[39] Lee E., Yoon Y. S., Kim D. J. (2018). Two-dimensional transition metal dichalcogenides and metal oxide hybrids for gas sensing. *ACS Sensors*.

[40] Wang J., Wei Y., Li H., Huang X., Zhang H. (2018). Crystal phase control in two-dimensional materials. *Science China-Chemistry*.

[41] Nguyen T. P., Choi S., Jeon J. M., Kwon K. C., Jang H. W., Kim S. Y. (2016). Transition metal disulfide nanosheets synthesized by facile sonication method for the hydrogen evolution reaction. *Journal of Physical Chemistry C*.

[42] Jung Y., Zhou Y., Cha J. J. (2016). Intercalation in two-dimensional transition metal chalcogenides. *Inorganic Chemistry Frontiers*.

[43] Yu J., Li J., Zhang W., Chang H. (2015). Synthesis of high quality two-dimensional materials via chemical vapor deposition. *Chemical Science*.

[44] Bhardwaj S. K., Bhardwaj N., Bhatt D., Malik P., Deep A. (2019). *Advances in the Synthesis and Development of Two-dimensional Transition-metal Dichalcogenide-based Nanosensor Platforms*.

[45] Li X., Shan J., Zhang W., Su S., Yuwen L., Wang L. (2017). Recent advances in synthesis and biomedical applications of two-dimensional transition metal dichalcogenide nanosheets. *Small*.

[46] Cushing B. L., Kolesnichenko V. L., O'Connor C. J. (2004). Recent advances in the liquid-phase syntheses of inorganic nanoparticles. *Chemical Reviews*.

[47] Chhowalla M., Liu Z., Zhang H. (2015). Two-dimensional transition metal dichalcogenide (TMD) nanosheets. *Chemical Society Reviews*.

[48] Yang S., Tongay S., Li Y. (2014). Layer-dependent electrical and optoelectronic responses of ReSe_2_ nanosheet transistors. *Nanoscale*.

[49] Gong C., Zhang Y., Chen W. (2017). Electronic and optoelectronic applications based on 2D novel anisotropic transition metal dichalcogenides. *Advanced Science*.

[50] Shim J., Park H. Y., Kang D. H. (2017). Electronic and optoelectronic devices based on two-dimensional materials: from fabrication to application. *Advanced Electronic Materials*.

[51] Voiry D., Yang J., Chhowalla M. (2016). Recent strategies for improving the catalytic activity of 2D TMD nanosheets toward the hydrogen evolution reaction. *Advanced Materials and Processes*.

[52] Zhou Z., Li B., Shen C. (2020). Metallic 1T phase enabling MoS_2_ Nanodots as an efficient agent for photoacoustic imaging guided photothermal therapy in the near-infrared-II window. *Small*.

[53] Pumera M., Sofer Z., Ambrosi A. (2014). Layered transition metal dichalcogenides for electrochemical energy generation and storage. *Journal of Materials Chemistry A*.

[54] Cho B., Hahm M. G., Choi M. (2015). Charge-transfer-based gas sensing using atomic-layer MoS_2_. *Scientific Reports*.

[55] Late D. J., Huang Y. K., Liu B. (2013). Sensing behavior of atomically thin-layered MoS_2_ transistors. *ACS Nano*.

[56] Jeong Y., Shin J., Hong Y. (2019). Gas sensing characteristics of the FET-type gas sensor having inkjet-printed WS_2_ sensing layer. *Solid-State Electronics*.

[57] Zhang S., Zhang W., Nguyen T. H., Jian J., Yang W. (2019). Synthesis of molybdenum diselenide nanosheets and its ethanol-sensing mechanism. *Materials Chemistry and Physics*.

[58] Hong Y., Kang W. M., Cho I. T., Shin J., Wu M., Lee J.-H. (2017). Gas-sensing characteristics of exfoliated WSe_2_ Field-Effect transistors. *Journal of Nanoscience and Nanotechnology*.

[59] Wu E., Xie Y., Yuan B. (2018). Specific and highly sensitive detection of ketone compounds based on p-type MoTe_2_ under ultraviolet illumination. *ACS Applied Materials & Interfaces*.

[60] Shim Y. S., Kwon K. C., Suh J. M. (2018). Synthesis of numerous edge sites in MoS_2_ via SiO_2_ Nanorods platform for highly sensitive gas sensor. *ACS Applied Materials & Interfaces*.

[61] Qin Z., Zeng D., Zhang J. (2017). Effect of layer number on recovery rate of WS_2_ nanosheets for ammonia detection at room temperature. *Applied Surface Science*.

[62] Kumar R., Goel N., Kumar M. (2018). High performance NO_2_ sensor using MoS_2_ nanowires network. *Applied Physics Letters*.

[63] Kathiravan D., Huang B. R., Saravanan A., Prasannan A., Hong P. D. (2019). Highly enhanced hydrogen sensing properties of sericin-induced exfoliated MoS_2_ nanosheets at room temperature. *Sensors and Actuators B: Chemical*.

[64] Pham T., Li G., Bekyarova E., Itkis M. E., Mulchandani A. (2019). MoS_2_-based optoelectronic gas sensor with sub-parts-per-billion limit of NO_2_ Gas detection. *ACS Nano*.

[65] Burman D., Ghosh R., Santra S., Guha P. K. (2016). Highly proton conducting MoS_2_/graphene oxide nanocomposite based chemoresistive humidity sensor. *RSC Advances*.

[66] Farahani H., Wagiran R., Hamidon M. N. (2014). Humidity sensors principle, mechanism, and fabrication technologies: a comprehensive review. *Sensors*.

[67] Li X., Li X., Li Z., Wang J., Zhang J. (2017). WS_2_ nanoflakes based selective ammonia sensors at room temperature. *Sensors and Actuators B: Chemical*.

[68] Asres G. A., Baldovi J. J., Dombovari A. (2018). Ultrasensitive H_2_S gas sensors based on p-type WS_2_ hybrid materials. *Nano Research*.

[69] Chhowalla M., Shin H. S., Eda G., Li L.-J., Loh K. P., Zhang H. (2013). The chemistry of two-dimensional layered transition metal dichalcogenide nanosheets. *Nature Chemistry*.

[70] Xu T., Liu Y., Pei Y. (2018). The ultra-high NO_2_ response of ultra-thin WS_2_ nanosheets synthesized by hydrothermal and calcination processes. *Sensors and Actuators B: Chemical*.

[71] Cho S.-Y., Kim S. J., Lee Y. (2015). Highly enhanced gas adsorption properties in vertically aligned MoS_2_ layers. *ACS Nano*.

[72] Zhao S., Xue J., Kang W. (2014). Gas adsorption on MoS_2_ monolayer from first-principles calculations. *Chemical Physics Letters*.

[73] Tabata H., Sato Y., Oi K., Kubo O., Katayama M. (2018). Bias- and gate-tunable gas sensor response originating from modulation in the Schottky barrier height of a Graphene/MoS_2_ van der Waals heterojunction. *ACS Applied Materials & Interfaces*.

[74] Feng Z., Chen B., Qian S. (2016). Chemical sensing by band modulation of a black phosphorus/molybdenum diselenide van der Waals hetero-structure. *2D Materials*.

[75] Hu Y., Zhou J., Yeh P. H., Li Z., Wei T. Y., Wang Z. L. (2010). Supersensitive, fast-response nanowire sensors by using Schottky contacts. *Advanced Materials*.

[76] Falak A., Tian Y., Yan L. (2019). Room temperature detection of NO_2_ at ppb level and full recovery by effective modulation of the barrier height for titanium oxide/graphene Schottky heterojunctions. *Advanced Materials Interfaces*.

[77] Kim S. J., Park J. Y., Yoo S. (2018). Carrier transport properties of MoS_2_ asymmetric gas sensor under charge transfer-based barrier modulation. *Nanoscale Research Letters*.

[78] Choi J., Kim Y. J., Cho S. Y. (2020). In situ formation of multiple Schottky barriers in a Ti_3_C_2_ MXene film and its application in highly sensitive gas sensors. *Advanced Functional Materials*.

[79] Mori T., Kozawa T., Ohwaki T. (1996). Schottky barriers and contact resistances on p‐type GaN. *Applied Physics Letters*.

[80] Cheng C. C., Wu C. L., Liao Y. M., Chen Y. F. (2016). Ultrafast and ultrasensitive gas sensors derived from a large Fermi-level shift in the Schottky junction with sieve-layer modulation. *ACS Applied Materials & Interfaces*.

[81] Kim Y., Kang S. K., Oh N. C. (2019). Improved sensitivity in Schottky contacted two-dimensional MoS_2_ gas sensor. *ACS Applied Materials & Interfaces*.

[82] Kim K., Kwak H. T., Cho H., Meyyappan M., Baek C. K. (2019). Design guidelines for high sensitivity ZnO nanowire gas sensors with Schottky contact. *IEEE Sensors Journal*.

[83] He Q., Zeng Z., Yin Z. (2012). Fabrication of flexible MoS_2_ thin-film transistor arrays for practical gas-sensing applications. *Small*.

[84] Zhou Z., Wang X., Zhang H. (2021). Activating layered metal oxide nanomaterials via structural engineering as biodegradable nanoagents for photothermal cancer therapy. *Small*.

[85] Ghatak S., Pal A. N., Ghosh A. (2011). Nature of electronic states in atomically thin MoS_2_ field-effect transistors. *ACS Nano*.

[86] Xu M., Liang T., Shi M., Chen H. (2013). Graphene-like two-dimensional materials. *Chemical Reviews*.

[87] Martin J., Akerman N., Ulbricht G. (2007). Observation of electron–hole puddles in graphene using a scanning single-electron transistor. *Nature Physics*.

[88] Li H., Yin Z., He Q. (2012). Fabrication of single- and multilayer MoS_2_ film-based field-effect transistors for sensing NO at room temperature. *Small*.

[89] Sharma B., Sharma A., Kim J.-S. (2018). Recent advances on H_2_ sensor technologies based on MOX and FET devices: a review. *Sensors and Actuators B: Chemical*.

[90] Haeng Yu J., Man Choi G. (1998). Electrical and CO gas sensing properties of ZnO-SnO_2_ composites. *Sensors and Actuators B: Chemical*.

[91] Wetchakun K., Samerjai T., Tamaekong N. (2011). Semiconducting metal oxides as sensors for environmentally hazardous gases. *Sensors and Actuators B: Chemical*.

[92] Mackin C., Fasoli A., Xue M. (2020). Chemical sensor systems based on 2D and thin film materials. *2D Materials*.

[93] Guillaud G., Simon J., Germain J. P. (1998). Metallophthalocyanines: Gas sensors, resistors and field effect transistors. *Coordination Chemistry Reviews*.

[94] Li Z., Zhao Q., Fan W., Zhan J. (2011). Porous SnO_2_ nanospheres as sensitive gas sensors for volatile organic compounds detection. *Nanoscale*.

[95] Kim J., Yong K. (2011). Mechanism study of ZnO nanorod-bundle sensors for H_2_S gas sensing. *Journal of Physical Chemistry C*.

[96] Hübner M., Simion C. E., Tomescu-Stănoiu A., Pokhrel S., Bârsan N., Weimar U. (2011). Influence of humidity on CO sensing with p-type CuO thick film gas sensors. *Sensors and Actuators B: Chemical*.

[97] Neri G. (2015). First fifty years of chemoresistive gas sensors. *Chemosensors*.

[98] Zhou X., Liu J., Wang C. (2015). Highly sensitive acetone gas sensor based on porous ZnFe_2_O_4_ nanospheres. *Sensors and Actuators B: Chemical*.

[99] Agmon N. (1995). The Grotthuss mechanism. *Chemical Physics Letters*.

[100] Zeng F. W., Liu X. X., Diamond D., Lau K. T. (2010). Humidity sensors based on polyaniline nanofibres. *Sensors and Actuators B: Chemical*.

[101] Ze L., Yueqiu G., Xujun L., Yong Z. (2017). MoS_2_-modified ZnO quantum dots nanocomposite: Synthesis and ultrafast humidity response. *Applied Surface Science*.

[102] Chen Z., Lu C. (2005). Humidity sensors: a review of materials and mechanisms. *Sensor Letters*.

[103] Yao M. S., Tang W. X., Wang G. E., Nath B., Xu G. (2016). MOF thin film-coated metal oxide nanowire array: significantly improved chemiresistor sensor performance. *Advanced Materials and Processes*.

[104] Ma N., Suematsu K., Yuasa M., Kida T., Shimanoe K. (2015). Effect of water vapor on Pd-loaded SnO_2_ nanoparticles gas sensor. *ACS Applied Materials & Interfaces*.

[105] Sangeetha M., Madhan D. (2020). Ultra sensitive molybdenum disulfide (MoS_2_)/graphene based hybrid sensor for the detection of NO_2_ and formaldehyde gases by fiber optic clad modified method. *Optics and Laser Technology*.

[106] Feo G., Sharma J., Kortukov D., Williams W., Ogunsanwo T. (2020). Distributed fiber optic sensing for real-time monitoring of gas in riser during offshore drilling. *Sensors*.

[107] Zhao C., Gan X., Yuan Q., Hu S., Fang L., Zhao J. (2018). High-performance volatile organic compounds microsensor based on few-layer MoS_2_-coated photonic crystal cavity. *Advanced Optical Materials*.

[108] Yang X., Chang A. S. P., Chen B., Gu C., Bond T. C. (2013). High sensitivity gas sensing by Raman spectroscopy in photonic crystal fiber. *Sensors and Actuators B: Chemical*.

[109] Kraeh C., Martinez-Hurtado J. L., Popescu A., Hedler H., Finley J. J. (2018). Slow light enhanced gas sensing in photonic crystals. *Optical Materials*.

[110] Zhou L., He B., Yang Y., He Y. (2014). Facile approach to surface functionalized MoS_2_ nanosheets. *RSC Advances*.

[111] Chou S. S., De M., Kim J. (2013). Ligand conjugation of chemically exfoliated MoS_2_. *Journal of the American Chemical Society*.

[112] Kim J. S., Yoo H. W., Choi H. O., Jung H. T. (2014). Tunable volatile organic compounds sensor by using thiolated ligand conjugation on MoS_2_. *Nano Letters*.

[113] Duy L. T., Kim D. J., Trung T. Q. (2015). High performance three-dimensional chemical sensor platform using reduced graphene oxide formed on high aspect-ratio micro-pillars. *Advanced Functional Materials*.

[114] Das S., Jayaraman V. (2014). SnO_2_: a comprehensive review on structures and gas sensors. *Progress in Materials Science*.

[115] Barzegar M., Berahman M., Iraji Zad A. (2018). Sensing behavior of flower-shaped MoS2nanoflakes: case study with methanol and xylene. *Beilstein Journal of Nanotechnology*.

[116] Li Y., Song Z., Li Y. (2019). Hierarchical hollow MoS_2_ microspheres as materials for conductometric NO_2_ gas sensors. *Sensors and Actuators B: Chemical*.

[117] Gu D., Li X., Wang H. (2018). Light enhanced VOCs sensing of WS_2_ microflakes based chemiresistive sensors powered by triboelectronic nangenerators. *Sensors and Actuators B: Chemical*.

[118] Prades J. D., Jimenez-Diaz R., Hernandez-Ramirez F. (2009). Equivalence between thermal and room temperature UV light-modulated responses of gas sensors based on individual SnO_2_ nanowires. *Sensors and Actuators B: Chemical*.

[119] Fan S. W., Srivastava A. K., Dravid V. P. (2009). UV-activated room-temperature gas sensing mechanism of polycrystalline ZnO. *Applied Physics Letters*.

[120] Park S., An S., Mun Y., Lee C. (2013). UV-enhanced NO_2_ gas sensing properties of SnO_2_-Core/ZnO-shell nanowires at room temperature. *ACS Applied Materials & Interfaces*.

[121] Liu L., Li X., Dutta P. K., Wang J. (2013). Room temperature impedance spectroscopy-based sensing of formaldehyde with porous TiO_2_ under UV illumination. *Sensors and Actuators B: Chemical*.

[122] Li X., Li X., Wang J., Lin S. (2015). Highly sensitive and selective room-temperature formaldehyde sensors using hollow TiO_2_ microspheres. *Sensors and Actuators B: Chemical*.

[123] Li X., Gao Y., Gong J., Zhang L., Qu L. (2009). Polyaniline/Ag composite nanotubes prepared through UV rays irradiation via fiber template approach and their NH_3_ Gas sensitivity. *Journal of Physical Chemistry C*.

[124] Leenaerts O., Partoens B., Peeters F. M. (2008). Adsorption ofH_2_O, NH_3_, CO, NO_2_, and NO on graphene: a first-principles study. *Physical Review B*.

[125] Ahmadi A., Beheshtian J., Hadipour N. L. (2011). Chemisorption of NH_3_ at the open ends of boron nitride nanotubes: a DFT study. *Structural Chemistry*.

[126] Tabata H., Matsuyama H., Goto T., Kubo O., Katayama M. (2021). Visible-light-activated response originating from carrier-mobility modulation of NO_2_ Gas sensors based on MoS_2_ monolayers. *ACS Nano*.

[127] Miller D. R., Akbar S. A., Morris P. A. (2014). Nanoscale metal oxide-based heterojunctions for gas sensing: a review. *Sensors and Actuators B: Chemical*.

[128] Bag A., Lee N. E. (2019). Gas sensing with heterostructures based on two-dimensional nanostructured materials: a review. *Journal of Materials Chemistry C*.

[129] Liu Q., Wang X., Wang J., Huang X. (2019). Spatially controlled two-dimensional heterostructures *via* solution-phase growth. *Acta Physico-Chimica Sinica*.

[130] Shao G., Xu Y., Liu S. (2019). Controllable preparation of 2D metal-semiconductor layered metal dichalcogenides heterostructures. *Science China-Chemistry*.

[131] Novoselov K. S., Mishchenko A., Carvalho A., Castro Neto A. H. (2016). 2D materials and van der Waals heterostructures. *Science*.

[132] Geim A. K., Grigorieva I. V. (2013). van der Waals heterostructures. *Nature*.

[133] Avalos-Ovando O., Mastrogiuseppe D., Ulloa S. E. (2019). Lateral heterostructures and one-dimensional interfaces in 2D transition metal dichalcogenides. *Journal of Physics: Condensed Matter*.

[134] Wu J., Peng J., Zhou Y. (2019). Solution processing for lateral transition-metal dichalcogenides homojunction from polymorphic crystal. *Journal of the American Chemical Society*.

[135] Friedman A. L., Perkins F. K., Hanbicki A. T., Culbertson J. C., Campbell P. M. (2016). Dynamics of chemical vapor sensing with MoS_2_ using 1T/2H phase contacts/channel. *Nanoscale*.

[136] Cho B., Kim A. R., Kim D. J. (2016). Two-dimensional atomic-layered alloy junctions for high-performance wearable chemical sensor. *ACS Applied Materials & Interfaces*.

[137] Wang X., Wang Z., Zhang J. (2018). Realization of vertical metal semiconductor heterostructures via solution phase epitaxy. *Nature Communications*.

[138] Yang K., Wang X., Li H. (2017). Composition- and phase-controlled synthesis and applications of alloyed phase heterostructures of transition metal disulphides. *Nanoscale*.

[139] Xiao Y., Zhou M., Liu J., Xu J., Fu L. (2019). Phase engineering of two-dimensional transition metal dichalcogenides. *Science China-Materials*.

[140] Sze S. M. (1969). *Physics of Semiconductor Devices*.

[141] Jha R. K., Guha P. K. (2018). Humidity sensing properties of coexfoliated heterogeneous WS_2_/WSe_2_ Nanohybrids. *IEEE Transactions on Nanotechnology*.

[142] Alhabeb M., Maleski K., Anasori B. (2017). Guidelines for synthesis and processing of two-dimensional titanium carbide (Ti_3_C_2_T_x_ MXene). *Chemistry of Materials*.

[143] Lipatov A., Alhabeb M., Lukatskaya M. R., Boson A., Gogotsi Y., Sinitskii A. (2016). Effect of synthesis on quality, electronic properties and environmental stability of individual monolayer Ti_3_C_2_ MXene flakes. *Advanced Electronic Materials*.

[144] Naguib M., Mochalin V. N., Barsoum M. W., Gogotsi Y. (2014). 25th anniversary article: MXenes: a new family of two-dimensional materials. *Advanced Materials*.

[145] Alhabeb M., Maleski K., Mathis T. S. (2018). Selective etching of silicon from Ti_3_SiC_2_ (MAX) to obtain 2D titanium carbide (MXene). *Angewandte Chemie International Edition*.

[146] Pang J., Mendes R. G., Bachmatiuk A. (2019). Applications of 2D MXenes in energy conversion and storage systems. *Chemical Society Reviews*.

[147] Liu G., Shen J., Liu Q. (2018). Ultrathin two-dimensional MXene membrane for pervaporation desalination. *Journal of Membrane Science*.

[148] Lv R., Robinson J. A., Schaak R. E. (2015). Transition metal dichalcogenides and beyond: synthesis, properties, and applications of single- and few-layer nanosheets. *Accounts of Chemical Research*.

[149] Ran J., Gao G., Li F.-T., Ma T.-Y., Du A., Qiao S.-Z. (2017). Ti_3_C_2_ MXene co-catalyst on metal sulfide photo-absorbers for enhanced visible-light photocatalytic hydrogen production. *Nature Communications*.

[150] Sinha A., Dhanjai, Zhao H. (2018). MXene: an emerging material for sensing and biosensing. *Trac-Trends in Analytical Chemistry*.

[151] Tan C., Cao X., Wu X. J. (2017). Recent advances in ultrathin two-dimensional nanomaterials. *Chemical Reviews*.

[152] Kim S. J., Koh H. J., Ren C. E. (2018). Metallic Ti_3_C_2_T_x_ MXene gas sensors with ultrahigh signal-to-noise ratio. *ACS Nano*.

[153] Naguib M., Mashtalir O., Carle J. (2012). Two-dimensional transition metal carbides. *ACS Nano*.

[154] Zhu J., Ha E., Zhao G. (2017). Recent advance in MXenes: a promising 2D material for catalysis, sensor and chemical adsorption. *Coordination Chemistry Reviews*.

[155] Sun Q., Wang J., Wang X. (2020). Treatment-dependent surface chemistry and gas sensing behavior of the thinnest member of titanium carbide MXenes. *Nanoscale*.

[156] Pazniak H., Plugin I. A., Loes M. J. (2020). Partially oxidized Ti_3_C_2_T_x_ MXenes for fast and selective detection of organic vapors at part-per-million concentrations. *ACS Applied Nano Materials*.

[157] Gantmakher V. F., Levinson Y. B., Grinberg A. A., Luryi S. (1988). Carrier scattering in metals and semiconductors. *Physics Today*.

[158] Lee E., VahidMohammadi A., Yoon Y. S., Beidaghi M., Kim D. J. (2019). Two-dimensional vanadium carbide MXene for gas sensors with ultrahigh sensitivity toward nonpolar gases. *ACS Sensors*.

[159] Liu F., Zhou A., Chen J. (2017). Preparation of Ti_3_C_2_ and Ti_2_C MXenes by fluoride salts etching and methane adsorptive properties. *Applied Surface Science*.

[160] Mashtalir O., Naguib M., Mochalin V. N. (2013). Intercalation and delamination of layered carbides and carbonitrides. *Nature Communications*.

[161] Lei J. C., Zhang X., Zhou Z. (2015). Recent advances in MXene: preparation, properties, and applications. *Frontiers of Physics*.

[162] Hope M. A., Forse A. C., Griffith K. J. (2016). NMR reveals the surface functionalisation of Ti_3_C_2_ MXene. *Physical Chemistry Chemical Physics*.

[163] Xiong D., Li X., Bai Z., Lu S. (2018). Recent advances in layered Ti_3_C_2_T_x_ MXene for electrochemical energy storage. *Small*.

[164] Zhang Y., Wang L., Zhang N., Zhou Z. (2018). Adsorptive environmental applications of MXene nanomaterials: a review. *RSC Advances*.

[165] Hantanasirisakul K., Gogotsi Y. (2018). Electronic and optical properties of 2D transition metal carbides and nitrides (MXenes). *Advanced Materials*.

[166] Berdiyorov G. R. (2015). Effect of surface functionalization on the electronic transport properties of Ti_3_C_2_ MXene. *Europhysics Letters*.

[167] Khazaei M., Arai M., Sasaki T. (2013). Novel electronic and magnetic properties of two-dimensional transition metal carbides and nitrides. *Advanced Functional Materials*.

[168] Khazaei M., Arai M., Sasaki T., Estili M., Sakka Y. (2014). Two-dimensional molybdenum carbides: potential thermoelectric materials of the MXene family. *Physical Chemistry Chemical Physics*.

[169] Khazaei M., Ranjbar A., Arai M., Sasaki T., Yunoki S. (2017). Electronic properties and applications of MXenes: a theoretical review. *Journal of Materials Chemistry C*.

[170] Zhou J., Khazaei M., Ranjbar A. (2019). Modulation of nearly free electron states in hydroxyl-functionalized MXenes: a first-principles study. *Journal of Materials Chemistry C*.

[171] Khazaei M., Ranjbar A., Ghorbani-Asl M. (2016). Nearly free electron states in MXenes. *Physical Review B*.

[172] Hajian S., Khakbaz P., Moshayedi M. Impact of different ratios of fluorine, oxygen, and hydroxyl surface terminations on Ti_3_C_2_T*_x_* MXene as ammonia sensor: a first-principles study.

[173] Yang D., Fang X., Zhao D., An Y., Hu Y., Luo Z. (2019). Sc_2_CO_2_ and Mn-doped Sc_2_CO_2_ as gas sensor materials to NO and CO: a first-principles study. *Physica E-Low-Dimensional Systems & Nanostructures*.

[174] Yang Z., Liu A., Wang C. (2019). Improvement of gas and humidity sensing properties of organ-like MXene by alkaline treatment. *ACS Sensors*.

[175] Zhou L., Zhang Y., Zhuo Z., Neukirch A. J., Tretiak S. (2018). Interlayer-decoupled Sc-based MXene with high carrier mobility and strong light-harvesting ability. *Journal of Physical Chemistry Letters*.

[176] Koh H. J., Kim S. J., Maleski K. (2019). Enhanced selectivity of MXene gas sensors through metal ion intercalation: in situ X-ray diffraction study. *ACS Sensors*.

[177] Chen W. Y., Jiang X., Lai S. N., Peroulis D., Stanciu L. (2020). Nanohybrids of a MXene and transition metal dichalcogenide for selective detection of volatile organic compounds. *Nature Communications*.

[178] Lee Y., Cho S. B., Chung Y. C. (2014). Tunable indirect to direct band gap transition of monolayer Sc_2_CO_2_ by the strain effect. *ACS Applied Materials & Interfaces*.

[179] Sahoo M. P. K., Wang J., Zhang Y., Shimada T., Kitamura T. (2016). Modulation of gas adsorption and magnetic properties of Monolayer-MoS_2_ by antisite defect and strain. *Journal of Physical Chemistry C*.

[180] Yu X., Li Y., Cheng J. (2015). Monolayer Ti_2_CO_2_: a promising candidate for NH_3_ sensor or capturer with high sensitivity and selectivity. *ACS Applied Materials & Interfaces*.

[181] Ma S., Yuan D., Jiao Z., Wang T., Dai X. (2017). Monolayer Sc_2_CO_2_: a promising candidate as a SO_2_ gas sensor or capturer. *Journal of Physical Chemistry C*.

[182] Li L. H. (2016). Effects of the interlayer interaction and electric field on the band gap of polar bilayers: a case study of Sc_2_CO_2_. *Journal of Physical Chemistry C*.

[183] Jia R., Xie P., Feng Y., Chen Z., Umar A., Wang Y. (2018). Dipole-modified graphene with ultrahigh gas sensibility. *Applied Surface Science*.

[184] Tang S., Cao Z. (2011). Adsorption of nitrogen oxides on graphene and graphene oxides: insights from density functional calculations. *Journal of Chemical Physics*.

[185] Yue Q., Shao Z., Chang S., Li J. (2013). Adsorption of gas molecules on monolayer MoS_2_ and effect of applied electric field. *Nanoscale Research Letters*.

[186] Yuan W., Yang K., Peng H., Li F., Yin F. (2018). A flexible VOCs sensor based on a 3D MXene framework with a high sensing performance. *Journal of Materials Chemistry A*.

[187] Bridgman P. W. (1914). Two new modifications of phosphorus. *Journal of the American Chemical Society*.

[188] Hanlon D., Backes C., Doherty E. (2015). Liquid exfoliation of solvent-stabilized few-layer black phosphorus for applications beyond electronics. *Nature Communications*.

[189] Liu H., Neal A. T., Zhu Z. (2014). Phosphorene: an unexplored 2D semiconductor with a high hole mobility. *ACS Nano*.

[190] Li L., Yu Y., Ye G. J. (2014). Black phosphorus field-effect transistors. *Nature Nanotechnology*.

[191] Carvalho A., Wang M., Zhu X., Rodin A. S., Su H., Castro Neto A. H. (2016). Phosphorene: from theory to applications. *Nature Reviews Materials*.

[192] Qiao J., Kong X., Hu Z. X., Yang F., Ji W. (2014). High-mobility transport anisotropy and linear dichroism in few-layer black phosphorus. *Nature Communications*.

[193] Debnath P. C., Park K., Song Y. W. (2018). Recent advances in black-phosphorus-based photonics and optoelectronics devices. *Small Methods*.

[194] Kong L., Qin Z., Xie G. (2016). Black phosphorus as broadband saturable absorber for pulsed lasers from 1*μ*m to 2.7*μ*m wavelength. *Laser Physics Letters*.

[195] Li L., Ye G. J., Tran V. (2015). Quantum oscillations in a two-dimensional electron gas in black phosphorus thin films. *Nature Nanotechnology*.

[196] Lee D., Choi Y., Hwang E., Kang M. S., Lee S., Cho J. H. (2016). Black phosphorus nonvolatile transistor memory. *Nanoscale*.

[197] Xia F., Wang H., Jia Y. (2014). Rediscovering black phosphorus as an anisotropic layered material for optoelectronics and electronics. *Nature Communications*.

[198] Low T., Roldán R., Wang H. (2014). Plasmons and screening in monolayer and multilayer black phosphorus. *Physical Review Letters*.

[199] Abbas A. N., Liu B., Chen L. (2015). Black phosphorus gas sensors. *ACS Nano*.

[200] Miao J., Zhang L., Wang C. (2019). Black phosphorus electronic and optoelectronic devices. *2D Materials*.

[201] Hu T., Han Y., Dong J. (2014). Mechanical and electronic properties of monolayer and bilayer phosphorene under uniaxial and isotropic strains. *Nanotechnology*.

[202] Kou L., Frauenheim T., Chen C. (2014). Phosphorene as a superior gas sensor: selective adsorption and distinct I-V response. *Journal of Physical Chemistry Letters*.

[203] Mahabal M. S., Deshpande M. D., Hussain T., Ahuja R. (2016). Sensing characteristics of phosphorene monolayers toward PH_3_ and AsH_3_ gases upon the introduction of vacancy defects. *Journal of Physical Chemistry C*.

[204] Yang A. J., Wang D. W., Wang X. H. (2017). Phosphorene: a promising candidate for highly sensitive and selective SF_6_ Decomposition gas sensors. *IEEE Electron Device Letters*.

[205] Cho S. Y., Lee Y., Koh H. J. (2016). Superior chemical sensing performance of black phosphorus: comparison with MoS_2_ and graphene. *Advanced Materials*.

[206] Tran V., Soklaski R., Liang Y., Yang L. (2014). Layer-controlled band gap and anisotropic excitons in few-layer black phosphorus. *Physical Review B*.

[207] Moon J., Park J. A., Lee S. J. (2013). A physicochemical mechanism of chemical gas sensors using an AC analysis. *Physical Chemistry Chemical Physics*.

[208] Mayorga-Martinez C. C., Sofer Z., Pumera M. (2015). Layered black phosphorus as a selective vapor sensor. *Angewandte Chemie International Edition*.

[209] Fattah A., Khatami S., Mayorga-Martinez C. C., Medina-Sánchez M., Baptista-Pires L., Merkoçi A. (2014). Graphene/silicon heterojunction Schottky diode for vapors sensing using impedance spectroscopy. *Small*.

[210] Snow E. S., Perkins F. K., Houser E. J., Badescu S. C., Reinecke T. L. (2005). Chemical detection with a single-walled carbon nanotube capacitor. *Science*.

[211] Lau C. N., Bao W., Velasco J. (2012). Properties of suspended graphene membranes. *Materials Today*.

[212] Cheng Z., Li Q., Li Z., Zhou Q., Fang Y. (2010). Suspended graphene sensors with improved signal and reduced noise. *Nano Letters*.

[213] Lee G., Kim S., Jung S., Jang S., Kim J. (2017). Suspended black phosphorus nanosheet gas sensors. *Sensors and Actuators B: Chemical*.

[214] Yang A., Wang D., Wang X., Zhang D., Koratkar N., Rong M. (2018). Recent advances in phosphorene as a sensing material. *Nano Today*.

[215] Erande M. B., Pawar M. S., Late D. J. (2016). Humidity sensing and photodetection behavior of electrochemically exfoliated atomically thin-layered black phosphorus nanosheets. *ACS Applied Materials & Interfaces*.

[216] Late D. J. (2016). Liquid exfoliation of black phosphorus nanosheets and its application as humidity sensor. *Microporous and Mesoporous Materials*.

[217] Cai Y., Ke Q., Zhang G., Zhang Y. W. (2015). Energetics, charge transfer, and magnetism of small molecules physisorbed on phosphorene. *Journal of Physical Chemistry C*.

[218] Yasaei P., Behranginia A., Foroozan T. (2015). Stable and selective humidity sensing using stacked black phosphorus flakes. *ACS Nano*.

[219] Lee G., Jung S., Jang S., Kim J. (2017). Platinum-functionalized black phosphorus hydrogen sensors. *Applied Physics Letters*.

[220] Suvansinpan N., Hussain F., Zhang G., Chiu C. H., Cai Y., Zhang Y.-W. (2016). Substitutionally doped phosphorene: electronic properties and gas sensing. *Nanotechnology*.

[221] Yuan Z., Li R., Meng F., Zhang J., Zuo K., Han E. (2019). Approaches to enhancing gas sensing properties: a review. *Sensors*.

[222] Chu B. H., Lo C. F., Nicolosi J. (2011). Hydrogen detection using platinum coated graphene grown on SiC. *Sensors and Actuators B: Chemical*.

[223] Chung M. G., Kim D. H., Seo D. K. (2012). Flexible hydrogen sensors using graphene with palladium nanoparticle decoration. *Sensors Actuators B: Chemical*.

[224] Cho S. Y., Koh H. J., Yoo H. W., Jung H. T. (2017). Tunable chemical sensing performance of black phosphorus by controlled functionalization with noble metals. *Chemistry of Materials*.

[225] Koenig S. P., Doganov R. A., Seixas L. (2016). Electron doping of ultrathin black phosphorus with Cu adatoms. *Nano Letters*.

[226] Kutlu E., Narin P., Lisesivdin S. B., Ozbay E. (2018). Electronic and optical properties of black phosphorus doped with Au, Sn and I atoms. *Philosophical Magazine*.

[227] Sun X., Luan S., Shen H., Lei S. (2018). Effect of metal doping on carbon monoxide adsorption on phosphorene: A first- principles study. *Superlattices and Microstructures*.

[228] Glavin N. R., Rao R., Varshney V. (2020). Emerging applications of elemental 2D materials. *Advanced Materials*.

[229] Xu S., Fu H., Tian Y. (2020). Exploiting two-dimensional Bi_2_O_2_Se for trace oxygen detection. *Angewandte Chemie International Edition*.

[230] Chandiramouli R. (2018). Antimonene nanosheet device for detection of explosive vapors - A first- principles inspection. *Chemical Physics Letters*.

[231] Shukla V., Warna J., Jena N. K., Grigoriev A., Ahuja R. (2017). Toward the realization of 2D borophene based gas sensor. *Journal of Physical Chemistry C*.

[232] Zhou X. F., Dong X., Oganov A. R., Zhu Q., Tian Y., Wang H.-T. (2013). Semimetallic two-dimensional boron allotrope with massless Dirac fermions. *Physical Review Letters*.

[233] Mannix A. J., Zhou X. F., Kiraly B. (2015). Synthesis of borophenes: anisotropic, two-dimensional boron polymorphs. *Science*.

[234] Liu L. Z., Xiong S. J., Wu X. L. (2016). Monolayer borophene electrode for effective elimination of both the Schottky barrier and strong electric field effect. *Applied Physics Letters*.

[235] Li W., Kong L., Chen C. (2018). Experimental realization of honeycomb borophene. *Science Bulletin*.

[236] Rubab A., Baig N., Sher M., Sohail M. (2020). Advances in ultrathin borophene materials. *Chemical Engineering Journal*.

[237] Nagarajan V., Chandiramouli R. (2017). Borophene nanosheet molecular device for detection of ethanol - A first- principles study. *Computational and Theoretical Chemistry*.

[238] Shahbazi Kootenaei A., Ansari G. (2016). B_36_ borophene as an electronic sensor for formaldehyde: quantum chemical analysis. *Physics Letters A*.

[239] Mohsenpour Z., Shakerzadeh E., Zare M. (2017). Quantum chemical description of formaldehyde (HCHO), acetaldehyde (CH_3_CHO) and propanal (CH_3_CH_2_CHO) pollutants adsorption behaviors onto the bowl-shaped B_36_ nanosheet. *Adsorption*.

[240] Omidvar A. (2017). Borophene: a novel boron sheet with a hexagonal vacancy offering high sensitivity for hydrogen cyanide detection. *Computational and Theoretical Chemistry*.

[241] Patel K., Roondhe B., Dabhi S. D., Jha P. K. (2018). A new flatland buddy as toxic gas scavenger: a first principles study. *Journal of Hazardous Materials*.

[242] Ye X. L., Lin S. J., Zhang J. W. (2021). Boosting room temperature sensing performances by atomically dispersed Pd stabilized via surface coordination. *ACS Sensors*.

[243] Potyrailo R. A. (2016). Multivariable sensors for ubiquitous monitoring of gases in the era of Internet of Things and industrial Internet. *Chemical Reviews*.

[244] Kumar R., Goel N., Hojamberdiev M., Kumar M. (2020). Transition metal dichalcogenides-based flexible gas sensors. *Sensors and Actuators A: Physical*.

[245] Peng C., Wei P., Chen X. (2018). A hydrothermal etching route to synthesis of 2D MXene (Ti_3_C_2_, Nb_2_C): enhanced exfoliation and improved adsorption performance. *Ceramics International*.

